# Variability in the Pre-Analytical Stages Influences Microbiome Laboratory Analyses

**DOI:** 10.3390/genes13061069

**Published:** 2022-06-15

**Authors:** Aditi Kumar, Kristin Gravdal, Jonathan P. Segal, Helen Steed, Matthew J. Brookes, Hafid O. Al-Hassi

**Affiliations:** 1 Department of Gastroenterology, The Royal Wolverhampton NHS Trust, Wolverhampton WV10 0QP, UK; aditikumar@nhs.net (A.K.); helen.steed@nhs.net (H.S.); matthew.brookes@nhs.net (M.J.B.); 2 Genetic Analysis AS, Kabelgata 8, 0580 Oslo, Norway; kristin.gravdal@genetic-analysis.com; 3 Department of Gastroenterology, Northern Hospital, Melbourne, VIC 3076, Australia; jonathansegal1@nhs.net; 4 School of Medicine and Clinical Practice, Faculty of Sciences and Engineering, University of Wolverhampton, Wolverhampton WV1 1LY, UK; 5 Research Institute in Healthcare Science, Faculty of Science and Engineering, University of Wolverhampton, Wolverhampton WV1 1LY, UK

**Keywords:** microbiome, pre-analysis, DNA, 16S rRNA

## Abstract

Introduction: There are numerous confounding variables in the pre-analytical steps in the analysis of gut microbial composition that affect data consistency and reproducibility. This study compared two DNA extraction methods from the same faecal samples to analyse differences in microbial composition. Methods: DNA was extracted from 20 faecal samples using either (A) chemical/enzymatic heat lysis (lysis buffer, proteinase K, 95 °C + 70 °C) or (B) mechanical and chemical/enzymatic heat lysis (bead-beating, lysis buffer, proteinase K, 65 °C). Gut microbiota was mapped through the 16S rRNA gene (V3–V9) using a set of pre-selected DNA probes targeting >300 bacteria on different taxonomic levels. Apart from the pre-analytical DNA extraction technique, all other parameters including microbial analysis remained the same. Bacterial abundance and deviations in the microbiome were compared between the two methods. Results: Significant variation in bacterial abundance was seen between the different DNA extraction techniques, with a higher yield of species noted in the combined mechanical and heat lysis technique (B). The five predominant bacteria seen in both (A) and (B) were *Bacteroidota* spp. and *Prevotella* spp. (*p =* NS), followed by *Bacillota* (*p =* 0.005), *Lachhnospiraceae* (*p =* 0.0001), *Veillonella* spp. (*p <* 0.0001) and *Clostridioides* (*p <* 0.0001). Conclusion: As microbial testing becomes more easily and commercially accessible, a unified international consensus for optimal sampling and DNA isolation procedures must be implemented for robustness and reproducibility of the results.

## 1. Introduction

An estimated 500–1000 species of bacteria exist in the human body at any one time with each bacterial strain accompanied by a genome consisting of thousands of genes [1]. The human gastrointestinal tract alone consists of 10–100 trillion symbiotic microbial cells that is encompassed by a single individual [2]. Traditionally, the intestinal microbiota composition has been evaluated using breath-testing techniques, small-bowel culture techniques, in situ hybridisation and culture-independent techniques. One of the most common approaches for microbiota profiling is targeting the 16S rRNA gene by using high-throughput next-generation sequencing (NGS) or probe-based analysis [3]. Although culturing techniques have improved in recent years with targeted phenotypic culturing, the majority of species cannot be cultured with standard methods [3,4]. The decreasing costs and increasing speed of DNA sequencing have prompted researchers to instead embark on small-subunit (16S) ribosomal RNA (rRNA) gene-sequence-based surveys of bacterial communities that reside on or in the human body [2]. NGS technologies have evolved over the past 15 years, becoming increasingly cost-effective and leading to substantial improvements in quality and yield [5], revolutionising genomics research by deciphering the complex microbial community from faecal samples which can be performed even in small laboratories [6]. The development of culture-independent DNA sequencing techniques has also allowed us to better understand the functionality of the microbiome, such as metagenomics, metabolomics, and meta-transcriptomics [7]. The variable regions (V1–V9) within the bacterial 16S rRNA genes show unique sequence diversity which can be utilised to determine bacterial taxa, sometimes at the species level [8]. Consequently, 16S rRNA sequencing has become the preferred method for studying microbial diversity [3].

Despite the recent advances in 16S rRNA sequencing, there are numerous host and environmental factors that can influence the gut microbiome. This includes patient factors, such as polypharmacy, history of antibiotic/pre- and probiotic use, age, gender, ethnicity, surgical history/comorbidities, smoking, geography and diet [9,10]. Whilst it may be difficult and unfeasible to standardise every one of these factors, optimising faecal collection, transportation, storage and DNA extraction techniques becomes essential to attain high-quality metagenomic DNA for an unbiased microbial analysis [9,11,12,13]. These numerous differences can all have variable impact on downstream results, making it difficult to directly compare results from different studies/patient cohorts/laboratories. As a result, there has been a lack of standardisation such that determining the causal role of the microbiota composition on disease pathogenesis has remained elusive. The primary aim of this study is to compare the pre-analytical steps, including handling of the samples pre-treatment and DNA extraction methods from the same human faecal samples between two different laboratory techniques and determine what, if any, differences can be seen in microbial composition. The outcome may aid in the standardisation of microbiome detection methods using 16S rRNA.

## 2. Materials and Methods

### 2.1. Ethical Approval and Good Clinical Practice

The study was performed in accordance with the recommendations guiding physicians in biomedical research involving human subjects, adopted by the 18th World Medical Assembly, Helsinki, Finland 1964, amended at Edinburgh in 2000. The study was conducted in accordance with the International Conference on Harmonisation Good Clinical Practice (ICH GCP) guidelines. Patient information was anonymised and any collection of patient data was in compliance of the Data Protection Act 1998. The study underwent full ethical approval by London—Stanmore Research Ethics Committee. REC ref: 16/LO/1325. All authors had access to the study data and reviewed and approved the final manuscript.

### 2.2. Human Samples

Faecal samples were collected from the Royal Wolverhampton NHS Trust, Wolverhampton, UK. Samples were collected from patients referred to gastroenterology for symptoms of diarrhoea. In total, 13 patients had confirmed bile acid diarrhoea (BAD) with a positive SeHCAT scan (<12% retention), of which 6 patients had a previous cholecystectomy, 3 had idiopathic BAD and 4 had a terminal ileal resection secondary to Crohn’s disease. The remaining 7 patients were diagnosed with functional diarrhoea and consisted of 1 patient with a previous cholecystectomy, 4 with no previous comorbidities and 2 with a terminal ileal resection secondary to Crohn’s disease (disease in remission). The faecal samples were collected prior to patients starting treatment for BAD and were excluded from the study if they received antibiotics within four weeks of the initial trial participation. Patients were given a large container and advised to store their first morning defecation into the tub. They were then advised to store the container in their fridge or freezer (if possible) or in a cold room such as the garage as the next possible option. The amount of stool obtained varied between patients and was collected from their home, office of work or hospital and each sample was then split into three universal tubes. An equal amount of stool was placed into each tube with no extra preservative added and stored in a −80 °C freezer on the same day of collection. The time variability between collection and storage was up to 8 h. A sample from one universal tube was used for DNA extraction in the Wolverhampton (WLV) laboratory (Method A), and the same sample from another tube was sent on dry ice to another laboratory for extraction, Genetic Analysis AS, Oslo, Norway (GA MAP) (Method B). For both laboratories, a single cycle of freeze/thaw occurred prior to DNA extraction and quantification.

### 2.3. DNA Extraction at Wolverhampton Laboratory (Method A)

Samples were analysed according to the manufacturer’s instruction using the commercially available QIAamp Fast DNA Stool Mini Kit (Qiagen, Manchester, UK). Briefly, Faecal samples were mixed with InhibitEX Buffer and homogenised by vortexing. Samples were incubated at 95 °C for 30 min. Samples were lysed with Protease K and Buffer AL prior to re-incubating at 70 °C for 10 min. The lysed samples were mixed with 100% ethanol and lysates, which was then loaded onto the QIAamp spin column. To remove any remaining impurities, the samples underwent two wash steps with buffers AW1 and AW2 and centrifuged. DNA was then eluted in low-salt buffer ATE. Purity and concentration of DNA were determined using the NanoDrop^TM^ 2000/2000c spectrophotometers (ThermoFisher, Manchester, UK) and an A260/280 ratio of 2.0 was accepted as ‘pure’ for DNA and A260/230 in the range of 0.9–1.2 considered free of other contaminants. A detailed protocol is available in the Supplementary Files.

### 2.4. DNA Extraction at GA Laboratory (Method B)

Faecal samples were extracted following the GA-map^®^ methods already published in detail in Casén et al. [3]. Briefly, each faecal sample was first homogenised before mixed with buffer (S.T.A.R., Roche, Basel, Switzerland). They were then transferred to Lysing Matrix E tubes (MP Biomedicals Inc., Santa Ana, CA, USA) and mechanically lysed in a FastPrep-96™ (MP Biomedicals Inc.). Lysed samples were centrifuged and incubated at 65 °C with lysis buffer and Protease K. Each protease-treated faecal sample was then used to extract total genomic DNA according to mag™ maxi kit instructions (LGC Genomics, Berlin, Germany), adjusted for a KingFisher^TM^ Flex 96/MagMAX™ Express-96 DNA extraction robot (Life Technologies, Waltham, MA, USA). This extraction method was extensively tested and validated in cooperation with the Norwegian University of Life Science (NMBU). The detailed protocol is provided in the Supplementary Files.

### 2.5. The GA-Map^®^ Technology Platform (GA-Map^®^ Dysbiosis Test)

WLV and GA MAP samples, extracted at the Wolverhampton or GA laboratory, respectively, were further analysed using the GA-map^®^ Dysbiosis Test Lx (Genetic Analysis AS, Oslo, Norway). The GA-map^®^ technology uses a pre-selected set of DNA probes (bacterial markers) and the variable regions of the bacterial 16S rRNA gene (V3 to V9) to map the intestinal microbiota and identify a bacterial profile.

Briefly, the polymerase chain reaction (PCR) was used to amplify ~1200 base pair fragments of the 16S rRNA gene (V3–V9), followed by a reaction clean-up as described previously [14]. The PCR template was then used in a probe labelling reaction (single-nucleotide extension), before hybridisation of the probe- and bead-set, fluorophore addition (Streptavidin-phycoerythrin) and detection, with the following modifications: a probe-set of 50 probes (48 bacterial target probes, a hybridisation control and universal control) was labelled, and hybridised to the GA-map^®^ bead set. The hybridisation signal was detected and quantified using a Luminex^®^ 200^TM^ instrument (Luminex Corp., Austin, TX, USA). Each of the probes in the GA-map^®^ test were designed to target a bacterial species or group based on their 16S rRNA sequence (V3–V9). Due to technical limitation in the Luminex instrument platform, six probes (probe no. 1, 15, 16, 26, 30, 49) were removed. The software then identified and quantified median signals, bead count and flags. Raw data was normalised before it was exported for further analysis. Additional information on the microbiome data analysis is provided in the Supplementary Files.

### 2.6. GA-Map^®^ Dysbiosis Test Analysis

This test was developed using a normal healthy cohort (n = 211) to build a normobiotic reference profile. This was then validated using an independent cohort of healthy individuals (n = 43) and patients with irritable bowel syndrome (n = 109) and inflammatory bowel disease (n = 135) [15]. This test is CE marked and suitable for use in a routine, clinical setting and in clinical research. This standardised protocol enables comparison of results between laboratories and between research studies, as well as for following patients over time. Based on the resulting normalised signal strengths, an algorithm compares the bacterial profile of the sample with that of a normal reference cohort and calculates the Dysbiosis Index (DI), ranging from 1 to 5. A DI value above 2 indicates a microbiota profile that differs from the normal reference population, and the higher the DI the further the bacterial profile deviates from the reference. Therefore, the higher the DI above 2, the more the sample is considered to deviate from normobiosis. Bacterial abundance is also calculated and is reported on a scale from −3 to +3 with 0 being the average level in the normal reference population. If the selected bacteria are reported to be less than 0, there is reduced abundance of that bacteria relative to the reference population, and if above 0, there is a greater abundance relative to the reference population.

### 2.7. Statistical Analysis

Data was analysed using Prism (Graphpad Version 9.2.0) and Microsoft Excel 2019 Version 16.55 (Microsoft, Redmond, WA, USA). Categorical data was expressed as the number of subjects (and percentage) and the mean, as appropriate. A test for association between the two techniques was performed using an independent *t*-test. The Mann–Whitney U test was used for testing DI values. All tests were two-sided and a *p*-value < 0.05 was considered to be statistically significant.

## 3. Results

A total of 20 patients equating to 40 samples were analysed (20 from method A and 20 from method B). The full details on patient demographics can be found in the Supplementary Files. The mean age of the patient cohort was 51.2 years with 60% being female. The median DNA concentration and interquartile range obtained from Method A was 16.3 ng/uL (IQR 12.9–20.9 ng/uL). The median DNA concentration from Method B was 17.8 ng/uL (14.9–19.6 ng/uL).

In total, 5 samples from method A and 1 sample from method B did not pass the GA-map^®^ Dysbiosis Test quality control requirements. Although method A is not validated for use with the Dysbiosis test, all the samples were still analysed for its microbiota.

**Figure 1:**
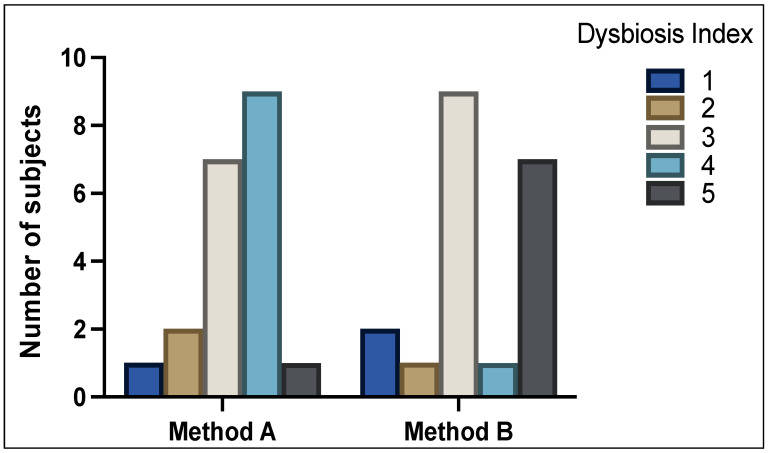
Distribution of Dysbiosis Index (DI) scores 1–5 as determined by GA-map^®^ Dysbiosis test. High variability of DI can be seen between method A and method B.

Figure 1 shows the DI scores between analysis of method A and method B samples (*p =* NS). With method A, 3 patients (15%) were classified as normobiotic (DI scale of 1 + 2), 7 patients (35%) with mild dysbiosis (DI scale of 3), and 10 patients (50%) with severe dysbiosis (DI scale of 4 + 5). With method B, 3 patients (15%) were classified as normobiotic (DI scale of 1 + 2), 9 patients (45%) with mild dysbiosis (DI scale of 3), and 8 patients (40%) showed severe dysbiosis (DI scale of 4 + 5).

**Figure 2:**
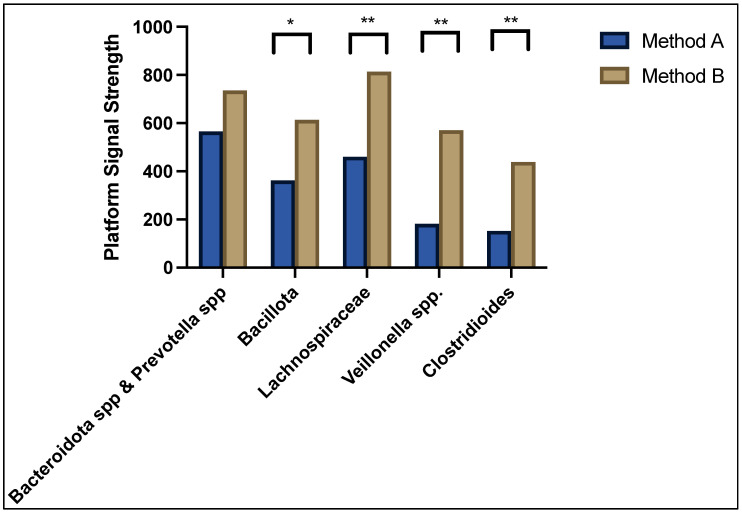
Median signal strength for top five predominant bacteria contributing to dysbiosis. * denotes *p* < 0.05 and ** denotes *p* < 0.001.

As can be seen in Figure 2, the predominant bacteria contributing to dysbiosis in both method A and B sample cohorts were *Bacteroidota* spp. and *Prevotella* spp. showing a normalised median platform signal strength of 565 and 735, respectively, *p =* NS; followed by *Bacillota* (362 vs. 614, *p =* 0.005), *Lachnospiraceae* (460 vs. 814, *p =* 0.0001), *Veillonella* spp. (182 vs. 570, *p <* 0.0001), and *Clostridioides* (152 vs. 439, *p <* 0.0001). The overall average abundance of bacterial composition for method A was 7.54 (95% confidence interval 0.0–21.5) and method B was 16.5 (95%CI 6.4–59.7), *p <* 0.0001. The differences in bacterial abundance as well as the full microbiota profile of each patient was compared between method A and method B and can be found in the Supplementary Files.

There are six phyla detected with the GA-map^®^ platform: *Actinomycetota, Bacteroidota, Bacillota, Pseudomonadota, Tenericutes* and *Verrucomicrobiota*. Out of the 48 bacterial probes used, 19 (39.6%) demonstrated statistically significant differences between methods A and B; 17/19 (89.5%) bacteria that had significant differences in abundance between the two methods were Gram positive bacteria.

**Figure 3:**
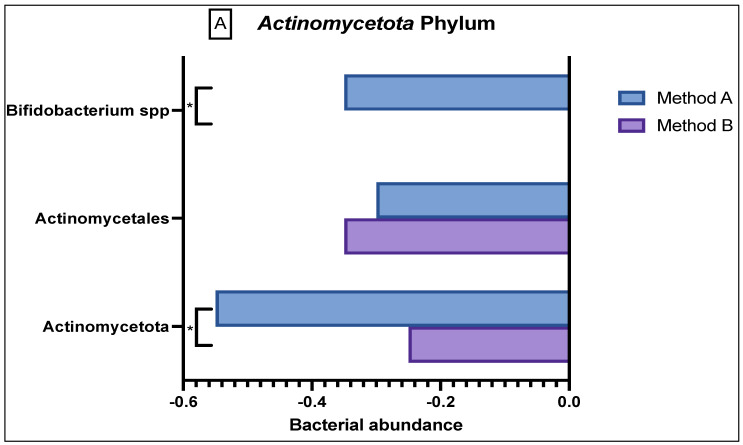

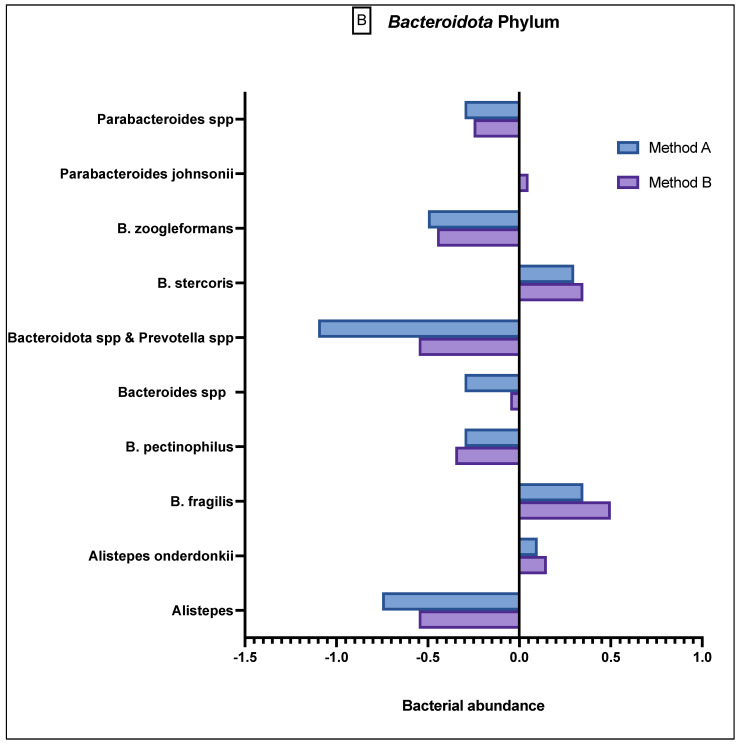

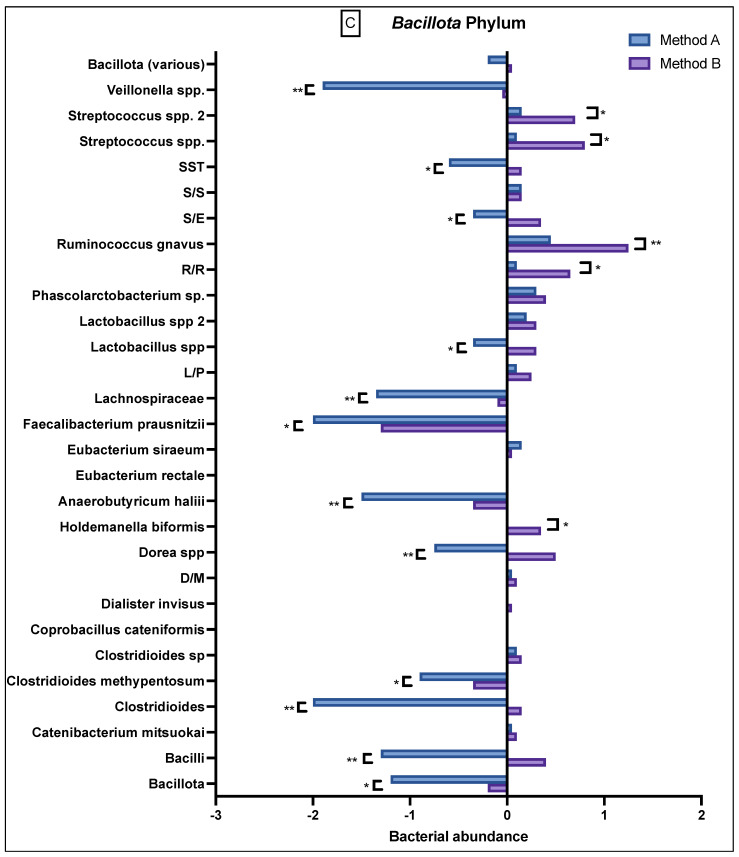

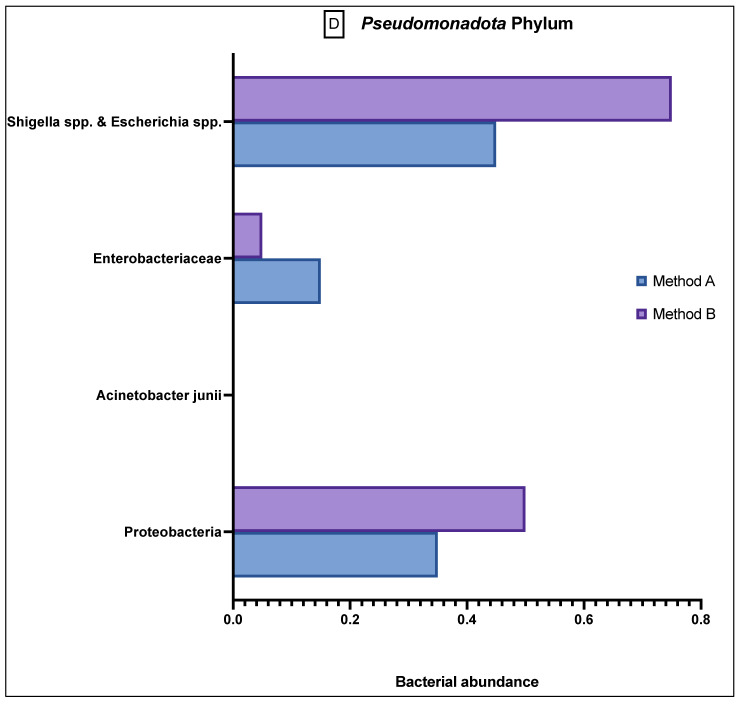

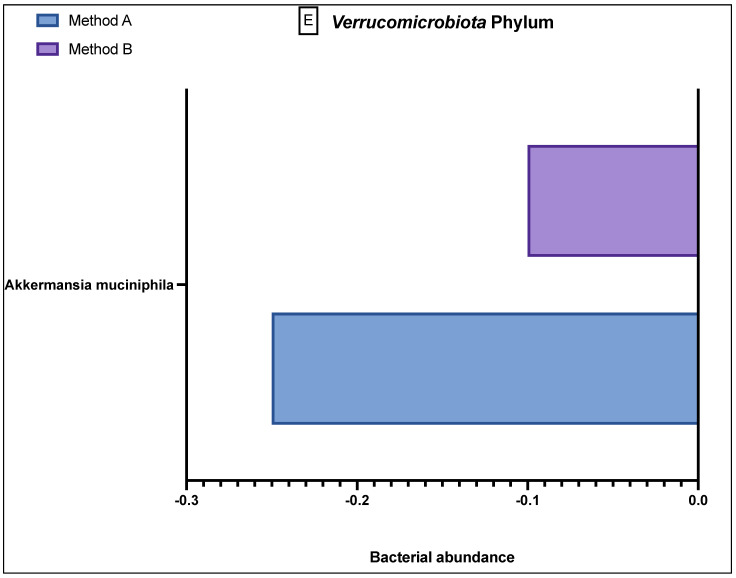
Comparison of bacterial abundance between method A and B samples as determined by the GA-map^®^ Dysbiosis test. Relative bacterial abundance is shown on a scale from ±3 in deviation from the GA-map^®^ normal reference range (0). Bacterial species are broken into their phyla: (**A**) *Actinomycetota*, (**B**) *Bacteroidota*, (**C**) *Bacillota*, (**D**) *Pseudomonadota*, (**E**) *Verrucomicrobiota*. The sixth phyla, *Tenericutes*, did not show any difference between the two methods and did not deviate from the normal reference range. D/M: *Dialister invisus* and *Megasphaera micronuciformis*, L/P: *Lactobacillus rumminus* and *Pediococcus acidolactici*, R/R: *Ruminococcus albus* and *R bromii*, S/E: *Streptococcus agalactiae* and *Eubacterium rectale*, S/S: *Streptococcus salivarius ssp thermophilus* and *Streptococcus sanguinis,* SST: *Streptococcus salivarius ssp thermophilus.* * denotes *p <* 0.05 and ** denotes *p <* 0.001.

In the *Actinomycetota* phylum (Figure 3A), there was reduced abundance seen of *Actinomycetales* and *Actinomycetota* in both the method A and B samples; however, there was a significant difference in abundance variation in the former (*p =* 0.03). Method A samples demonstrated a reduced abundance in *Bifidobacteria* as compared to the sample from method B (*p =* 0.0047).

The Bacteroidota phylum (Figure 3B) demonstrated generally reduced abundance in the species *Alistepes*, *B pectinophilus*, *Bacteroidota* spp., *Bacteroidota* spp. and *Prevotella* spp., *B zoogleformans* and *Parabacteroides* spp., and a greater abundance in *Alistepes onderdonkii*, *B fragilis*, *B stercoris* and *Parabacteroides jonsonii*. Between the two methods, the results demonstrated generally similar abundance levels.

The *Bacillota* phylum (Figure 3C) had variable abundance, but there were notable differences seen between A and B. Specifically, method B samples consistently demonstrated a greater abundance in *Bacilli, Clostridia*, *Dialister invisus* and *Megasphaera micronuciformis (D/M), Lactobacillus* spp., *Streptococcus agalactiae* and *Eubacterium rectale (S/E), Streptococcus salivarius ssp thermophilus (SST),* and *Bacillota* (various), whilst the method A samples demonstrated a reduced abundance in these bacteria. In contrast, method A demonstrated a higher reduced abundance of *Veillonella*, *Streptococcus salivarius ssp thermophilus*, *Streptococcus agalactiae* and *Eubacterium rectale*, *Lactobacillus* spp., *Lachnospiraceae*, *Faecalibacterium prausnitzii, Anaerobutyricum halii, Dorea* spp., *Clostridioides methypentosum, Clostridioides, Bacilli* and *Bacillota*. Apart from *Bacillota* (various), these differences were statistically significant.

The *Pseudomonadota* phylum (Figure 3D) generally showed a greater abundance than the relative population in both A and B samples, and although there was still variation between the different bacteria, the results were not statistically significant.

The *Verrucomicrobiota* phylum (Figure 3E) demonstrated only one prominent bacterium, *Akkermansia muciniphilia*, which showed a greater reduced abundance in the method A samples as compared to method B. The final phylum, *Tenericutes*, which had one relevant bacterial species, *Mycoplasma hominis*, is not shown in Figure 3 because in all 40 samples, this bacterium was found to be in the reference population range, with no variation in abundance in either the method A or B samples.

## 4. Discussion

This study demonstrated significant variation in bacterial abundance in 19/48 bacterial probes due to methodological differences in faecal DNA extraction, with the majority of the differences seen in Gram-positive bacteria. The primary difference in DNA extraction between the method A and B samples was the combined use of mechanical and chemical/enzymatic heat lysis in the latter whilst only using chemical/enzymatic heat lysis in the former. There are two main DNA extraction methods, which are mechanical lysis/bead-beating and chemical lysis. Whilst bead-beating is considered to produce superior DNA yields, bacterial diversity, Gram-positive bacteria, spores and fungi [16,17], vigorous bead-beating should be avoided due to risk of shearing the nucleic acids [18,19] which can lead to the formation of chimeric molecules during PCR amplification [20,21]. Whilst emulsion PCR may help to prevent chimera formation, this was not used in our study and future work using this technique should be considered. Although recent studies have shown that chemical lysis is not inferior to mechanical lysis in rumen samples and from human saliva [16,22], human gut samples have consistently shown that mechanical lysis or a combination of enzymatic and mechanical disruption results in higher degrees of microbial diversity and have the greatest effect on gut microbiome composition [17,23,24,25]. Furthermore, our results demonstrated that between the two methods, there was considerable variation in relative abundances within Gram-positive bacteria but similar abundance levels in the Gram-negative Bacteroidota phylum. It is well known that due to their thick, rigid cell walls, Gram-positive microorganisms are resistant to cell lysis, which can be overcome through the use of bead-beating [26]. Thus, these findings corroborate previous studies confirming that a combination technique is of greater use in detecting and isolating Gram-positive bacteria [17,22,27].

Besides the use of bead-beating or mechanical lysis, another difference between the two protocols includes the initial step of homogenising, or thoroughly mixing of the stool prior to subsampling. Previous studies have demonstrated that there can be high variability of gut microbes within a single faecal sample [28] and homogenisation can reduce within-stool heterogeneity [12,29]. Hsieh et al. also demonstrated that homogenised stools were more similar to each other than samples that were not homogenised, with a general increase seen in phyla-containing Gram-positive bacteria [29]. Whilst this mixing step was performed in method B, it was not a part of the DNA extraction protocol from method A, highlighting again the need to standardise the sample treatment in the pre-analytical steps.

A confounding factor that could have affected the results and interpretation of this study is the handling of the faecal samples after collection. Changes in temperature and humidity can alter or contaminate the samples [30], as can transit conditions and duration of travel. The microbial composition is unstable from the point of sample collection and thus immediate freezing at −80 °C is advised to conserve microbial diversity, which can be significantly altered by dry storage at 4 °C [31]. Whilst our samples were stored at −80 °C immediately after collection, there were delays in receiving the patients’ samples from the time of defecation by up to 8 h. Some studies have reported that optimal freezing time should be completed within 15 min of defecation [12] whilst others suggest within 2 h [32], although the feasibility of doing this is questionable. Furthermore, multiple studies have shown that repeated temperature fluctuations or dramatic temperature changes are a major stress for bacteria, leading to DNA aggravation and degradation [33,34,35]. It is, therefore, particularly important to avoid repeated freeze–thaw cycles. Whilst we attempted to minimise the number of freeze–thaw cycles, transportation from the hospital to the lab and from the lab to Norway was unavoidable and was done with the use of dry ice. The transfers from the hospital to the lab was a short journey of 10 minutes and samples were kept on dry ice and were immediately stored in a −80 °C freezer on arrival. The samples were also kept on dry ice to ensure that the temperature was consistent during the period of travel between the countries. Therefore, we maintained minimal (to none) freeze–thaw cycles, indicating that our microbial results were a true outcome rather than possible variability from sample storage and transport.

The technique used to map bacterial targets by GA-map^®^ with the use of DNA probes are pre-determined and pre-defined on certain taxonomic levels; therefore, it is difficult to comment whether there was variation seen between the two methods in extracting different bacterial species and demonstrating greater microbial diversity. The use of NGS may have provided a more comprehensive analysis, allowing for a broader range of microbial groups on each taxonomic level. However, probe-based analysis has several advantages including an easily understandable, semi-quantitative score of dysbiosis (based on bacterial abundance and profile within a sample), a standardised protocol which enables comparison of results between laboratories and between research projects, and the test is CE marked making it suitable for use in a routine clinical setting as well as in clinical research. The primary aim of our study was to demonstrate variability in results when using different pre-analytical techniques and whether we used NGS or probe-based analysis should not change our final results as there was clear variability in bacterial abundance seen between the two pre-analytical protocols. The general low signal strengths and low abundance levels in method A, which used only chemical/enzymatic heat lysis indicates that the bacterial target extraction was not (as) successful as method B, which used the combination of mechanical and chemical/enzymatic heat lysis techniques.

Ultimately, the major limitation to this study is the small sample size. In spite of this, our study still demonstrated significant variability in the microbial composition when all parameters apart from the pre-analytical DNA extraction technique remained the same. Whilst it may be difficult to regulate inter-individual variables such as age, gender and other environmental factors, the pre-analytical inter-assay methodology needs to be standardised to reduce variability in microbial analysis results. Several different extraction methods have been published and proven to work well to identify the microbiome using faecal samples. However, standardisation and the validation of methods is the most important to ensure comparable results over time and between different laboratories and studies. As microbial testing becomes more easily and commercially accessible, developing a unified international standard and consensus for the pre-analytical stages will become essential to ensure robustness and reproducibility of the results. Otherwise, there will always remain a fundamental bias generated from certain DNA extraction protocols when comparing microbiota datasets. As shown in our study, differences in extraction methods affects bacterial abundance and this should be taken into consideration when interpreting study findings reported in the literature.

## Data Availability

Requests for any data, analytic methods and study materials will be considered and made available upon request to the corresponding author. Individual participant data will not be shared.

## Supplementary Materials

The following supporting information can be downloaded at: https://www.mdpi.com/article/10.3390/genes13061069/s1, Supplementary Method A: DNA extraction at Wolverhampton laboratory; Supplementary Method B: DNA extraction at GA laboratory; Figure S1: Comparison of platform signal strength between Method A and B across all 48 bacterial probes; Figure S2: The following twenty heat graphs display the differences in bacterial abundance (demonstrated by platform signal strength) of each patient’s microbial profile between Method A and Method B; Table S1: List of the bacterial targets of the 48 probes in the GA-map® Dysbiosis Test Lx; Table S2: Patient demographics of the twenty patients with bile acid diarrhoea.

## References

[1] Gilbert J.A., Blaser M.J., Caporaso J.G., Jansson J.K., Lynch S.V., Knight R. (2018). Current understanding of the human microbiome. Nat. Med..

[2] Turnbaugh P.J., Ley R.E., Hamady M., Fraser-Liggett C.M., Knight R., Gordon J.I. (2007). The human microbiome project. Nature.

[3] Casen C., Vebo H.C., Sekelja M., Hegge F.T., Karlsson M.K., Ciemniejewska E., Dzankovic S., Froyland C., Nestestog R., Engstrand L. (2015). Deviations in human gut microbiota: A novel diagnostic test for determining dysbiosis in patients with IBS or IBD. Aliment. Pharm. Ther..

[4] Browne H.P., Forster S.C., Anonye B.O., Kumar N., Neville B.A., Stares M.D., Goulding D., Lawley T.D. (2016). Culturing of ‘unculturable’ human microbiota reveals novel taxa and extensive sporulation. Nature.

[5] Goodwin S., McPherson J.D., McCombie W.R. (2016). Coming of age: Ten years of next-generation sequencing technologies. Nat. Rev. Genet..

[6] van Dijk E.L., Auger H., Jaszczyszyn Y., Thermes C. (2014). Ten years of next-generation sequencing technology. Trends Genet..

[7] Mullish B.H., Quraishi M.N., Segal J.P., Ianiro G., Iqbal T.H. (2021). The gut microbiome: What every gastroenterologist needs to know. Frontline Gastroenterol..

[8] Allaband C., McDonald D., Vazquez-Baeza Y., Minich J.J., Tripathi A., Brenner D.A., Loomba R., Smarr L., Sandborn W.J., Schnabl B. (2019). Microbiome 101: Studying, Analyzing, and Interpreting Gut Microbiome Data for Clinicians. Clin. Gastroenterol. Hepatol..

[9] Murgas Torrazza R., Neu J. (2011). The developing intestinal microbiome and its relationship to health and disease in the neonate. J. Perinatol..

[10] Tasnim N., Abulizi N., Pither J., Hart M.M., Gibson D.L. (2017). Linking the Gut Microbial Ecosystem with the Environment: Does Gut Health Depend on Where We Live?. Front. Microbiol..

[11] Wu W.-K., Chen C.-C., Panyod S., Chen R.-A., Wu M.-S., Sheen L.-Y., Chang S.-C. (2019). Optimization of fecal sample processing for microbiome study—The journey from bathroom to bench. J. Formos. Med. Assoc..

[12] Gorzelak M.A., Gill S.K., Tasnim N., Ahmadi-Vand Z., Jay M., Gibson D.L. (2015). Methods for Improving Human Gut Microbiome Data by Reducing Variability through Sample Processing and Storage of Stool. PLoS ONE.

[13] Voigt A.Y., Costea P.I., Kultima J.R., Li S.S., Zeller G., Sunagawa S., Bork P. (2015). Temporal and technical variability of human gut metagenomes. Genome Biol..

[14] Vebo H.C., Sekelja M., Nestestog R., Storro O., Johnsen R., Oien T., Rudi K. (2011). Temporal development of the infant gut microbiota in immunoglobulin E-sensitized and nonsensitized children determined by the GA-map infant array. Clin. Vaccine Immunol..

[15] Wei S., Bahl M.I., Baunwall S.M.D., Hvas C.L., Licht T.R. (2021). Determining Gut Microbial Dysbiosis: A Review of Applied Indexes for Assessment of Intestinal Microbiota Imbalances. Appl. Env. Microbiol..

[16] Ma Z.Y., Zhang X.M., Wang R., Wang M., Liu T., Tan Z.L. (2020). Effects of Chemical and Mechanical Lysis on Microbial DNA Yield, Integrity, and Downstream Amplicon Sequencing of Rumen Bacteria and Protozoa. Front. Microbiol..

[17] de Boer R., Peters R., Gierveld S., Schuurman T., Kooistra-Smid M., Savelkoul P. (2010). Improved detection of microbial DNA after bead-beating before DNA isolation. J. Microbiol. Methods.

[18] Bharti R., Grimm D.G. (2021). Current challenges and best-practice protocols for microbiome analysis. Brief. Bioinfor..

[19] Yu Z., Morrison M. (2004). Improved extraction of PCR-quality community DNA from digesta and fecal samples. Biotechniques.

[20] Von Wintzingerode F., Gobel U.B., Stackebrandt E. (1997). Determination of microbial diversity in environmental samples: Pitfalls of PCR-based rRNA analysis. FEMS Microbiol. Rev..

[21] Zhang B., Brock M., Arana C., Dende C., van Oers N.S., Hooper L.V., Raj P. (2021). Impact of Bead-Beating Intensity on the Genus- and Species-Level Characterization of the Gut Microbiome Using Amplicon and Complete 16S rRNA Gene Sequencing. Front. Cell Infect. Microbiol..

[22] Li X., Bosch-Tijhof C.J., Wei X., de Soet J.J., Crielaard W., Loveren C.V., Deng D.M. (2020). Efficiency of chemical versus mechanical disruption methods of DNA extraction for the identification of oral Gram-positive and Gram-negative bacteria. J. Int. Med. Res..

[23] Lim M.Y., Song E.J., Kim S.H., Lee J., Nam Y.D. (2018). Comparison of DNA extraction methods for human gut microbial community profiling. Syst. Appl. Microbiol..

[24] Karasartova D., Gureser A.S., Ruh E., Turegun-Atasoy B., Calgin M.K., Tasci L., Taylan-Ozkan A. (2018). An alternative DNA extraction method for detection of Blastocystis spp. in human fecal samples. Exp. Parasitol..

[25] Videnska P., Smerkova K., Zwinsova B., Popovici V., Micenkova L., Sedlar K., Budinska E. (2019). Stool sampling and DNA isolation kits affect DNA quality and bacterial composition following 16S rRNA gene sequencing using MiSeq Illumina platform. Sci. Rep..

[26] Auer G.K., Weibel D.B. (2017). Bacterial Cell Mechanics. Biochemistry.

[27] Hwang K.Y., Kwon S.H., Jung S.O., Lim H.K., Jung W.J., Park C.S., Kim J.H., Suh K.Y., Huh N. (2011). Miniaturized bead-beating device to automate full DNA sample preparation processes for gram-positive bacteria. Lab Chip.

[28] Human Microbiome Project (2012). A framework for human microbiome research. Nature.

[29] Hsieh Y.H., Peterson C.M., Raggio A., Keenan M.J., Martin R.J., Ravussin E., Marco M.L. (2016). Impact of Different Fecal Processing Methods on Assessments of Bacterial Diversity in the Human Intestine. Front. Microbiol..

[30] Thomas T., Gilbert J., Meyer F. (2012). Metagenomics—A guide from sampling to data analysis. Microb. Inf. Exp..

[31] Choo J.M., Leong L.E., Rogers G.B. (2015). Sample storage conditions significantly influence faecal microbiome profiles. Sci. Rep..

[32] Yeoh Y.K., Chen Z., Hui M., Wong M.C.S., Ho W.C.S., Chin M.L., Ng S.C., Chan F.K.L., Chan P.K.S. (2019). Impact of inter- and intra-individual variation, sample storage and sampling fraction on human stool microbial community profiles. PeerJ.

[33] Cuthbertson L., Rogers G.B., Walker A.W., Oliver A., Hafiz T., Hoffman L.R., Carroll M.P., Parkhill J., Bruce K.D., van der Gast C.J. (2014). Time between collection and storage significantly influences bacterial sequence composition in sputum samples from cystic fibrosis respiratory infections. J. Clin. Microbiol..

[34] Cardona S., Eck A., Cassellas M., Gallart M., Alastrue C., Dore J., Azpiroz F., Roca J., Guarner F., Manichanh C. (2012). Storage conditions of intestinal microbiota matter in metagenomic analysis. BMC Microbiol..

[35] Tedjo D.I., Jonkers D.M., Savelkoul P.H., Masclee A.A., van Best N., Pierik M.J., Penders J. (2015). The effect of sampling and storage on the fecal microbiota composition in healthy and diseased subjects. PLoS ONE.

