# Supplementary material for: Variability in the Pre-Analytical Stages Influences Microbiome Laboratory Analyses
Source: Genes (Basel). 2022 Jun 15;13(6):1069. doi: 10.3390/genes13061069 (PMC9223004; doi:10.3390/genes13061069)
Supplement: Supplementary file 1 [file genes-13-01069-s001.zip › genes-1716530-supplementary.pdf]

Supplementary Methods:

DNA extraction at Wolverhampton laboratory (Method A)

Samples were analysed according to the manufacturer's instruction using the commercially available QIAamp Fast DNA Stool Mini Kit, provided by Qiagen. Briefly, 180-220mg of stool was weighed and placed in a 1.5ml centrifuge tube. 1ml of InhibitEX Buffer was added to each stool sample and vortexed until faecal matter was thoroughly homogenised. Samples were then placed in a hot water bath at 95°C for 30 minutes. Once removed from the water bath, samples were vortexed for 15 seconds and then centrifuged for 1 minute at 14000rpm at room temperature. The stool particles formed a pellet, of which 200µl supernatant was transferred to a new microcentrifuge tube which had 15µl of proteinase K. 200µl of Buffer AL was placed into the same tube and the sample was vortexed to ensure thorough mixing of the solution. The samples were then incubated in a hot water bath at 70°C for 10 minutes. Once removed from the water bath, 200µl of 100% ethanol was added to the lysate and vortexed again. The sample was then transferred to a QIAamp spin column and centrifuged for 1 minute. The filtrate was discarded. 500µl of Buffer AW1 was added to the QIAamp spin column and centrifuged for 1 minute. The filtrate was discarded. 500µl of Buffer AW2 was added to the QIAamp spin column and centrifuged for 3 minutes. The filtrate was discarded and the QIAamp spin column was centrifuged for a further 3 minutes. The QIAamp spin column was transferred into a new microcentrifuge tube and 70µl of Buffer ATE was added directly into the QIAamp membrane. The samples were incubated at room temperature for 1 minute and then centrifuged for 1 minute to elute the DNA. Purity and concentration of DNA were determined using the NanoDrop™ 2000/2000c Spectrophotometers (ThermoFisher) and an A260/280 ratio of 2.0 was accepted as 'pure' for DNA and A260/230 in the range of 0.9-1.2 considered free of other contaminants.

DNA extraction at GA laboratory (Method B)

Faecal samples were mixed with stool transport and recovery buffer (Roche, Basel, Switzerland) in a 1:3 ratio by vortexing. All samples were pulse centrifuged and 600 µl was transferred to a 96-well Lysing Matrix E rack (MP Biomedicals Inc., Santa Ana, CA, USA). Samples were mechanically lysed twice at 1800 rpm, 40s on 40s rest, in a FastPrep-96™ (MP Biomedicals Inc.). Lysed samples were centrifuged (5 min, 1300g, PlateSpin II centrifuge, Kubota, Tokyo, Japan), and 250µl was incubated at 65°C for 15 min with 250µl lysis buffer

BLM and 20 µl protease. A 400µl aliquot of each protease-treated faecal sample was used to extract total genomic DNA according to mag™ maxi kit instructions (LGC Genomics, Berlin, Germany), adjusted for a MagMAX™ express 96 DNA extraction robot (Life Technologies, Waltham, MA, USA).

Single nucleotide extension (SNE), hybridisation and detection.

The PCR template (>75ng) was used in a single-nucleotide extension (SNE) reaction described in Vebø et al [1], with the following modifications: a final volume of 25µl containing 0.5µM BIOTIN-11-ddCTP (Perkin Elmer, Waltham, MA, USA) was used through five labelling cycles to label a probe-set of 50 probes (0.01 µM) (48 bacteria target probes, a hybridisation control and Universal control) (Supplementary table 1). Complementary probes coupled to carboxylated barcoded magnetic beads (Luminex) were hybridised to the SNE probes and quantified using a Luminex® 200™ (Luminex). In brief, a 10µl SNE sample was added to a 40µl reaction volume containing 31.2µl hybridization buffer, hybridisation control and 1.8µl coupled beads. Samples were incubated at 700 rpm, 95°C for 3 min, followed by 700 rpm, 45°C for 15 min in a Vortemp™ 56 (Labnet International Inc., Edison, NJ, USA). A 25µl aliquot of buffer containing 20µg/ml streptavidin R-phycoerythrin LumiGrade ultrasensitive reagent (Roche) was added to each sample before 90 minutes of incubation at 700 rpm, 45°C. Finally, samples were washed. The hybridisation signal was processed by the Luminex® 200™ xPONENT® software (Luminex). The software identified and quantified median signals, bead count and flags, and raw data files were exported for further analysis.

Probe number	Phylum	Class	Genus
1	Actinomycetota	Actinomycetota	*Bifidobacterium*
2	Actinomycetota	Actinomycetota	
3	Actinomycetota	Actinomycetota	
4	Bacteroidota	Bacteroidia	*Alistipes*
5	Bacteroidota	Bacteroidia	*Alistipes*
6	Bacteroidota	Bacteroidia	*Bacteroides*
7	Bacteroidota	Bacteroidia	*Bacteroides/Prevotella*

8	Bacteroidota	Bacteroidia	*Bacteroides*
9	Bacteroidota	Bacteroidia	*Bacteroides*
10	Bacteroidota	Bacteroidia	*Bacteroides*
11	Bacteroidota	Bacteroidia	*Bacteroides*
12	Bacteroidota	Bacteroidia	*Parabacteroides*
13	Bacteroidota	Bacteroidia	*Parabacteroides*
14	Bacillota	Bacilli	*Lactobacillus*
15	Bacillota	Bacilli	*Lactobacillus*
16	Bacillota	Bacilli	*Pedicoccus/Lactobacillus*
17	Bacillota	Bacilli	*Streptococcus*
18	Bacillota	Bacilli	*Streptococcus*
19	Bacillota	Bacilli	*Streptococcus*
20	Bacillota	Bacilli	*Streptococcus*
21	Bacillota	Bacilli	
22	Bacillota	Bacilli/Clostridia	*Streptococcus/Eubacterium*
23	Bacillota	Clostridia	*Blautia*
24	Bacillota	Clostridia	*Clostridium*
25	Bacillota	Clostridia	*Clostridium*
26	Bacillota	Clostridia	*Dorea*
27	Bacillota	Clostridia	*Eubacterium*
28	Bacillota	Clostridia	*Eubacterium*
29	Bacillota	Clostridia	*Eubacterium*
30	Bacillota	Clostridia	*Faecalibacterium*
31	Bacillota	Clostridia	*Ruminococcus*
32	Bacillota	Clostridia	
33	Bacillota	Clostridia	
34	Bacillota	Erysipelotrichia	*Catenibacterium*
35	Bacillota	Erysipelotrichia	*Coprobacillus*
36	Bacillota	Erysipelotrichia	Unclassified Erysipelotrichaceae

37	Bacillota	Negativicutes	*Dialister*
38	Bacillota	Negativicutes	*Megasphaera/Dialister*
39	Bacillota	Negativicutes	*Phascolarctobacterium*
40	Bacillota	Negativicutes	
41	Bacillota	Negativicutes/ Epsilonproteobacteria/ Clostridia	*Veillonella/Helicobacter*
42	Bacillota/ Tenericutes/ Bacteroidota species		
43	Pseudomonadota	Gammaproteobacteria	*Acinetobacter*
44	Pseudomonadota	Gammaproteobacteria	*Salmonella, Citrobacter, Cronobacter, Enterobacter*
45	Pseudomonadota	Gammaproteobacteria	*Shigella/Escherichia*
46	Pseudomonadota		
47	Tenericutes	Mollicutes	*Mycoplasma*
48	Verrucomicrobia	Verrucomicrobiae	*Akkermansia*

Supplementary Table S1: List of the bacterial targets of the 48 probes in the GA-map® Dysbiosis Test Lx

		Total (n=20)
Age (years)		49.95
Gender		
	Male	8 (40%)
	Female	12 (60%)
Ethnicity		
	White British	20 (100%)
Co-morbidities		
	Ischaemic heart disease	0
	Diabetes Mellitus	2 (10%)
	Dyspepsia/Gastro-oesophageal reflux disease	1 (5%)
	Malignancy	1 (5%)
	Ulcerative colitis	0
	Crohn's disease	7 (35%)
	Anxiety/Depression	2 (10%)
	Chronic pain/ Fibromyalgia	1 (5%)
	Others[a]	6 (30%)
	Nil	4 (20%)
	Unknown	1 (5%)
Medications		
	Anti-depressants[b]	4 (20%)
	Proton Pump Inhibitors	6 (30%)
	Anti-spasmodics	3 (15%)
	Analgesics[c]	3 (15%)
	Biologics	2 (10%)
	Immunosuppressants	2 (10%)
	Others[d]	6 (30%)
	Nil	6 (30%)
	Unknown	1 (5%)

Supplementary Table S2: Patient demographics of the twenty patients with bile acid diarrhoea.

- *a- Includes hypertension, cerebrovascular disease, asthma or chronic obstructive pulmonary disease, hypercholesterolaemia, epilepsy, and hypothyroid disease.*
- *b- Although generally prescribed for depression and/or anxiety, some patients were prescribed this for analgesic relief.*
- *c- Includes co-codamol or other opioid derivative, non-steroidal anti-inflammatory agents, pregabalin or gabapentin.*
- *d- Includes hypoglycaemics, anti-hypertensives, anti-epileptics, statins*

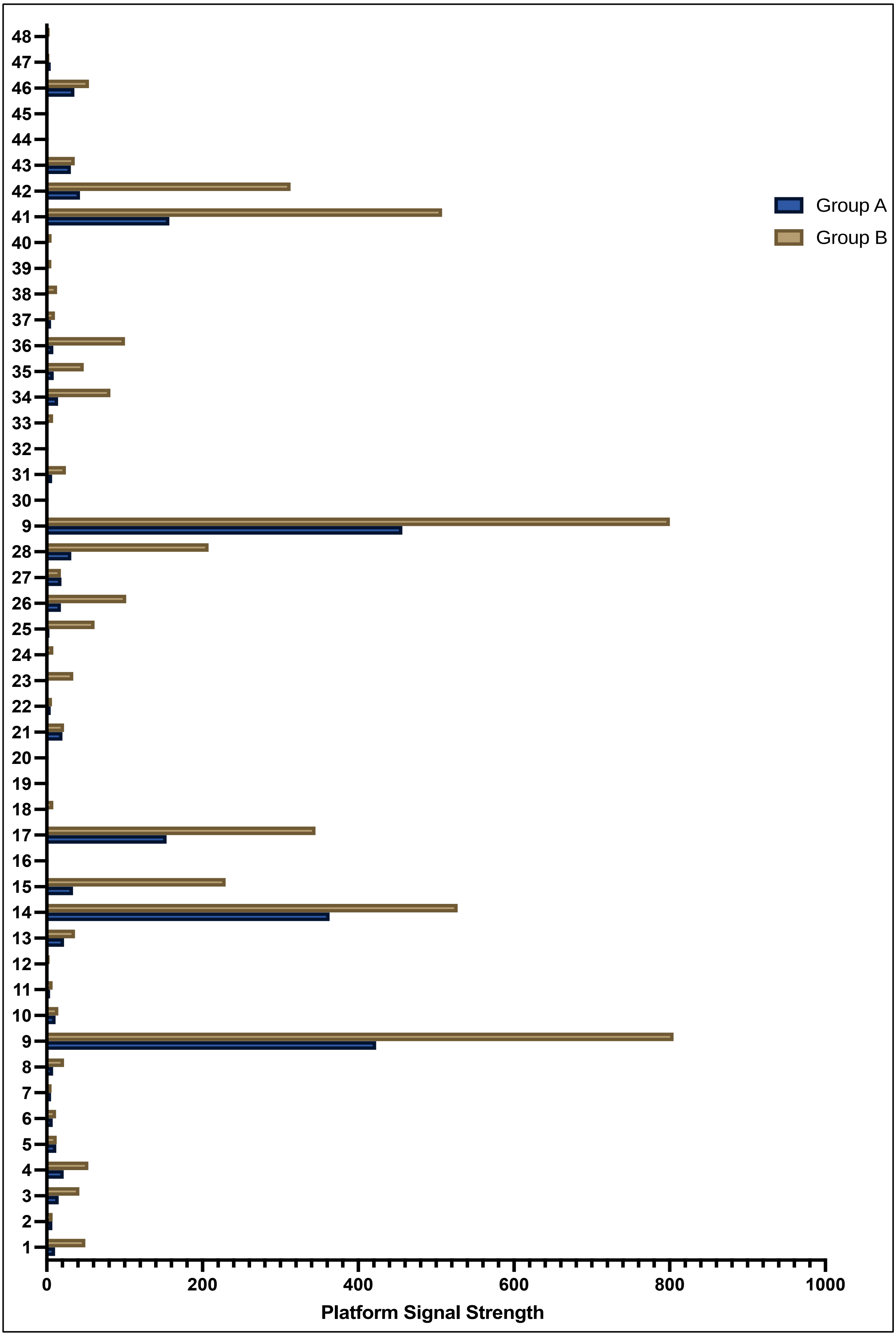

48
47
46
45
44
43
42
41
40
39
38
37
36
35
34
33
32
31
30
9
28
27
26
25
24
23
22
21
20
19
18
17
16
15
14
13
12
11
10
9
8
7
6
5
4
3
2
1
0
200
400
600
800
1000
Platform Signal Strength
Group A
Group B

Supplementary Figure S1: Comparison of platform signal strength between Method A and B across all 48 bacterial probes; p<0.0001. 1= *Actinobacteria*, 2= A*ctinomycetales*, 3=*Bifidobacterium spp*, 4=*Alistipes*, 5= *Alistipes onderdonkii*, 6= *Bacteroides fragilis*, 7= *Bacteroides pectinophilus*, 8= *Bacteroides spp*, 9= *Bacteroides spp* & *Prevotella spp*, 10= *Bacteroides stercoris*, 11= *Bacteroides zoogleoformans*, 12= *Parabacteroides jonsonii*, 13= *Parabacteroides spp*, 14= *Firmicutes*, 15= *Bacilli*, 16= *Catenibacterium mitsuokai*, 17= *Clostridia*, 18= *Clostridium methylpentosum*, 19= *Clostridium sp*., 20= *Coprobacillus cateniformis*, 21 = *Dialister invisus*, 22= *Dialister invisus* & *Megasphaera micronuciformis*, 23= *Dorea spp*, 24= *Eubacterium biforme*, 25= *Eubacterium hallii*, 26= *Eubacterium rectale*, 27= *Eubacterium siraeum*, 28= *Faecalibacterium prasunitzii*, 29= *Lachnospiraceae*, 30= *Lactobacillus ruminis* & *Pediococcus acidilactici*, 31= *Lactobacillus spp*, 32= *Lactobacillus spp 2*, 33= *Phascolarctoba cterium sp*, 34= *Ruminococcus albus* & *Ruminococcus bromii*, 35= *Ruminococcus gnavus*, 36= *Streptococcus agalactiae* & *Eubacterium rectale*, 37= *Streptococcus salivarius ssp thermophilus* & *Streptococcus sanguinis*, 38= *Streptococcus salivarius ssp thermophilus*, 39= *Streptococcus spp*, 40= *Streptococcus spp 2*, 41= *Veillonella spp*, 42= *Firmicutes (various)*, 43= *Proteobacteria*, 44= *Acinetobacter junii*, 45= *Enterobacteriaceae*, 46= *Shigella spp* & *Escherichia spp*, 47= *Mycoplasma hominis*, 48= *Akkermansia muciniphila*

Supplementary Figure S2: The following twenty heat graphs display the differences in bacterial abundance (demonstrated by platform signal strength) of each patient's microbial profile between Method A and Method B. Statistical analysis was performed using the Wilcoxon test to determine (if) any significant differences between Method A and B. 1= *Actinobacteria*, 2= Act*inomycetales*, 3=*Bifidobacterium spp*, 4=*Alistipes*, 5= *Alistipes onderdonkii*, 6= *Bacteroides fragilis*, 7= *Bacteroides pectinophilus*, 8= *Bacteroides spp*, 9= *Bacteroides spp* & *Prevotella spp*, 10= *Bacteroides stercoris*, 11= *Bacteroides zoogleoformans*, 12= *Parabacteroides jonsonii*, 13= *Parabacteroides spp*, 14= *Firmicutes*, 15= *Bacilli*, 16= *Catenibacterium mitsuokai*, 17= *Clostridia*, 18= *Clostridium methylpentosum*, 19= *Clostridium sp*., 20= *Coprobacillus cateniformis*, 21 = *Dialister invisus*, 22= *Dialister invisus* & *Megasphaera micronuciformis*, 23= *Dorea spp*, 24= *Eubacterium biforme*, 25= *Eubacterium hallii*, 26= *Eubacterium rectale*, 27= *Eubacterium siraeum*, 28= *Faecalibacterium prasunitzii*, 29= *Lachnospiraceae*, 30= *Lactobacillus ruminis* & *Pediococcus acidilactici*, 31= *Lactobacillus spp*, 32= *Lactobacillus spp 2*, 33= *Phascolarctoba cterium sp*, 34= *Ruminococcus albus* & *Ruminococcus bromii*, 35= *Ruminococcus gnavus*, 36= *Streptococcus agalactiae* & *Eubacterium rectale*, 37= *Streptococcus salivarius ssp thermophilus* & *Streptococcus sanguinis*, 38= *Streptococcus salivarius ssp thermophilus*, 39= *Streptococcus spp*, 40= *Streptococcus spp 2*, 41= *Veillonella spp*, 42= *Firmicutes (various)*, 43= *Proteobacteria*, 44= *Acinetobacter junii*, 45= *Enterobacteriaceae*, 46= *Shigella spp* & *Escherichia spp*, 47= *Mycoplasma hominis*, 48= *Akkermansia muciniphila.*

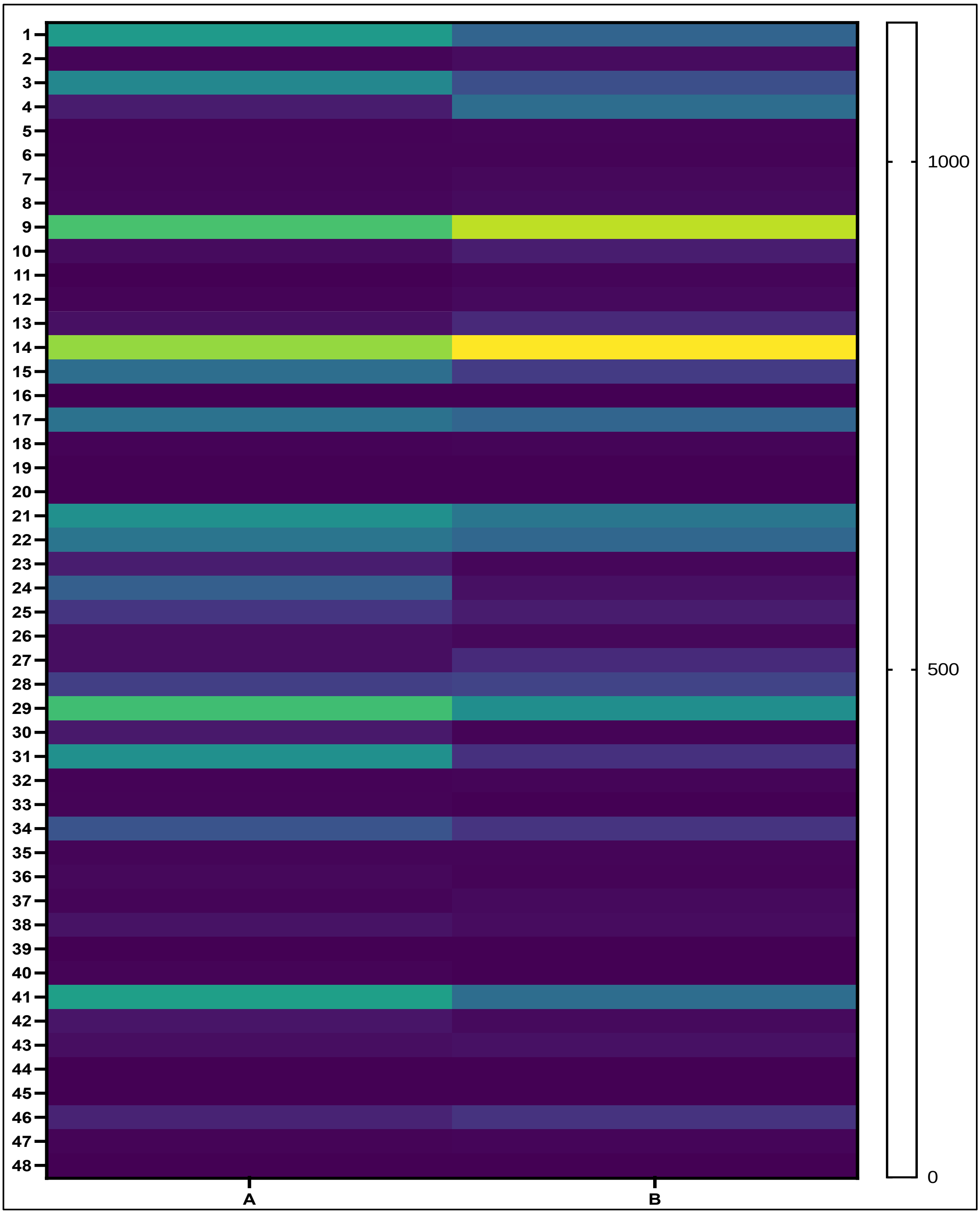

Patient 1. P = 0.96

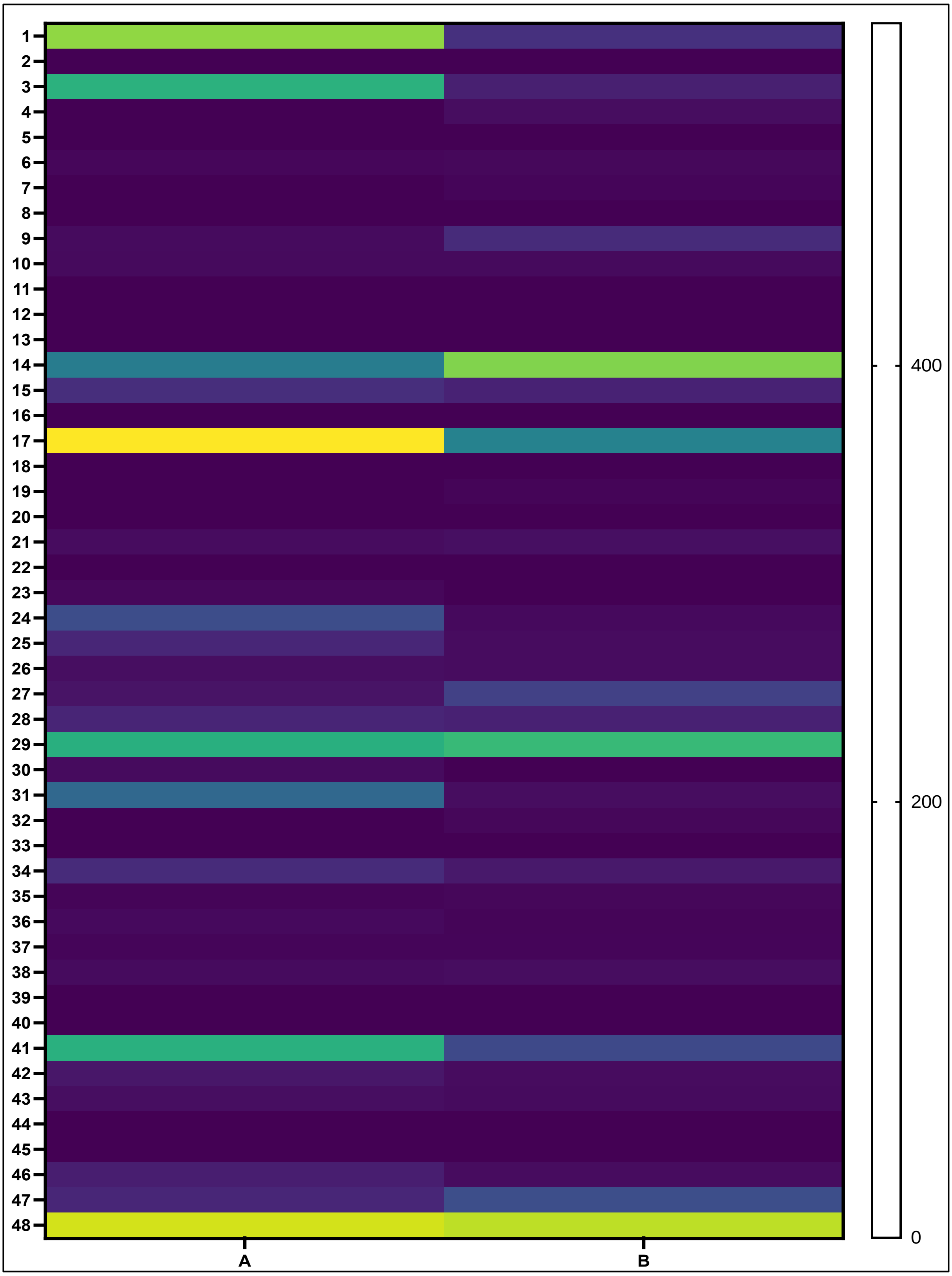

Patient 2. P < 0.0001

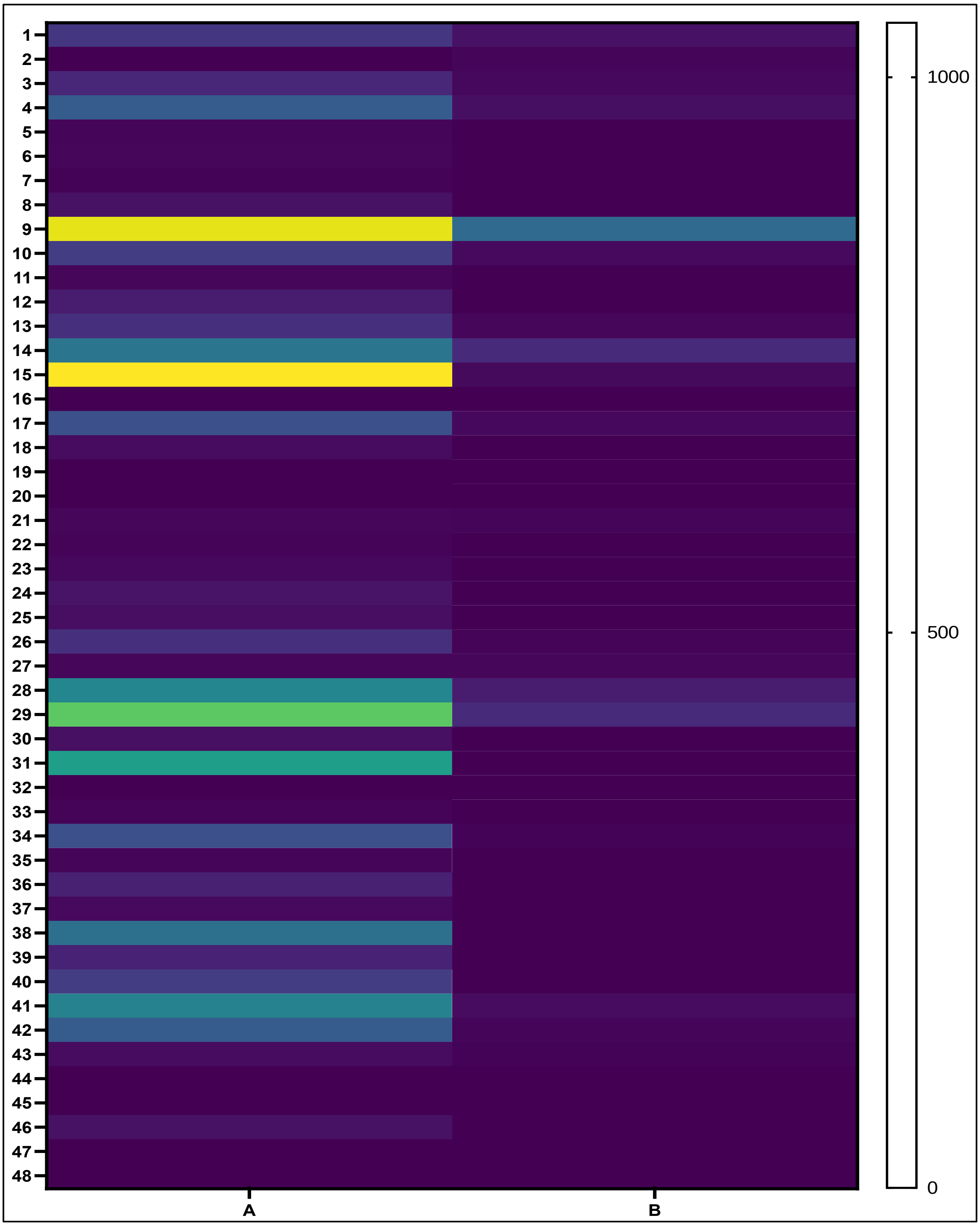

Patient 3. P < 0.0001

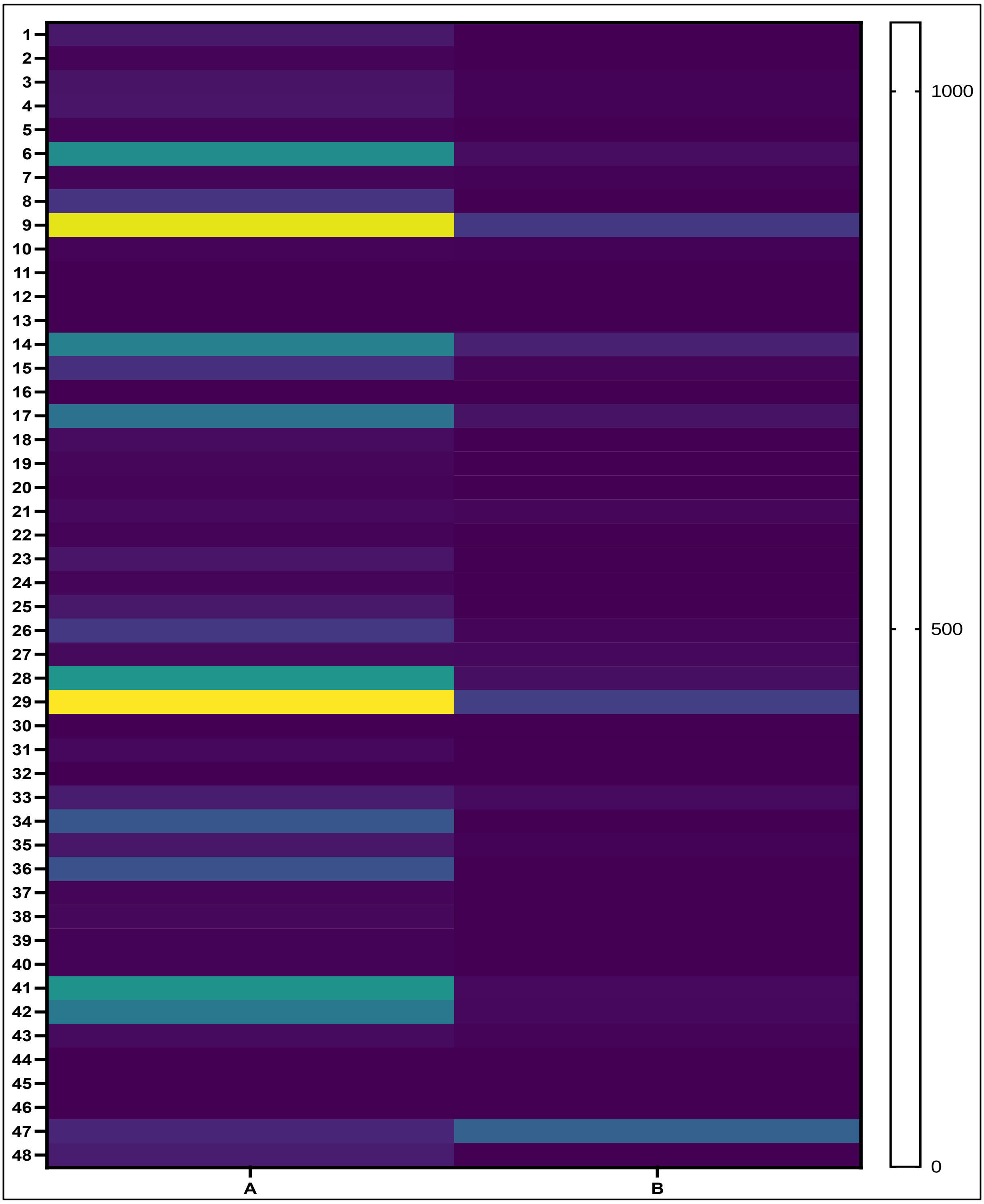

Patient 4: P < 0.0001

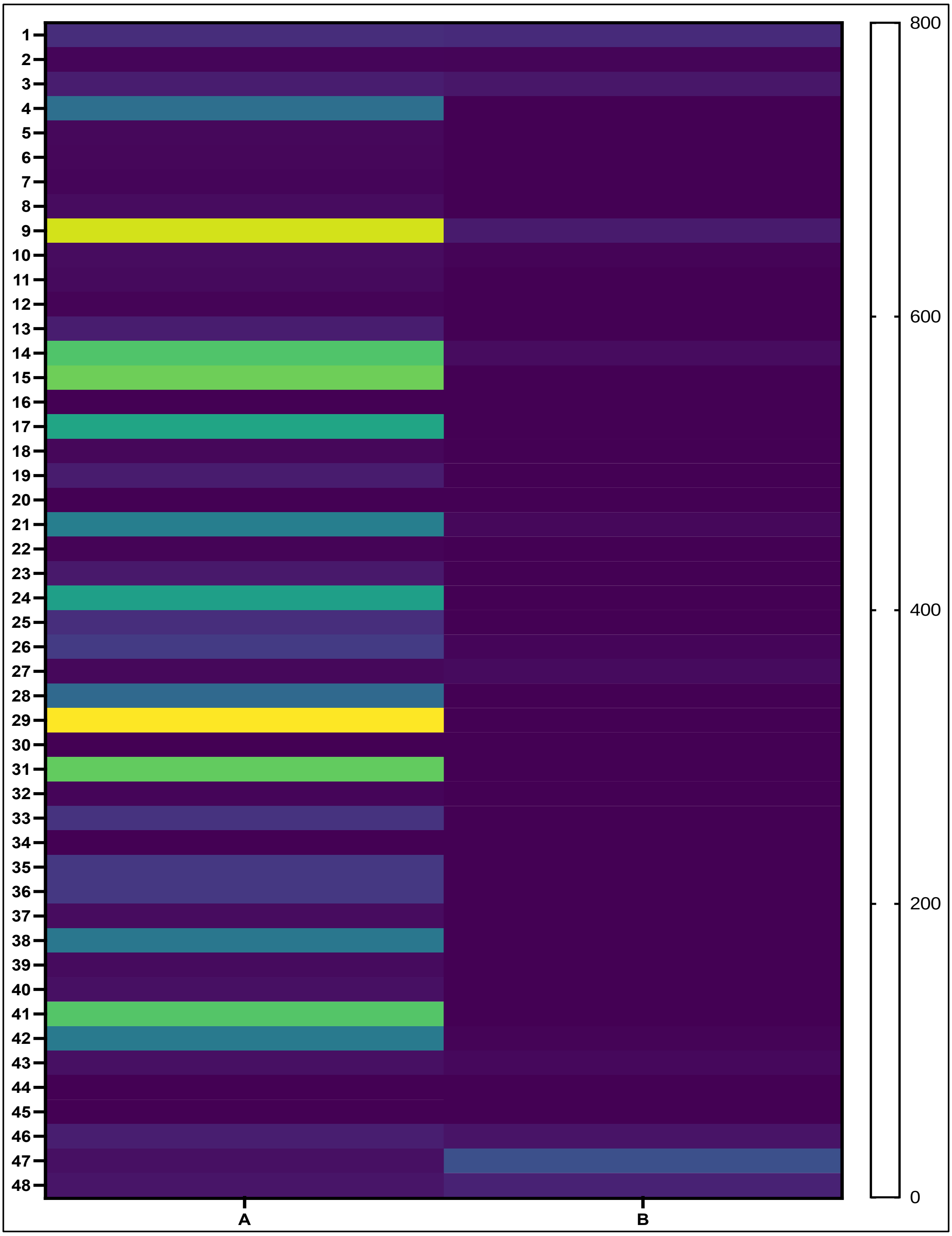

Patient 5. P < 0.0001

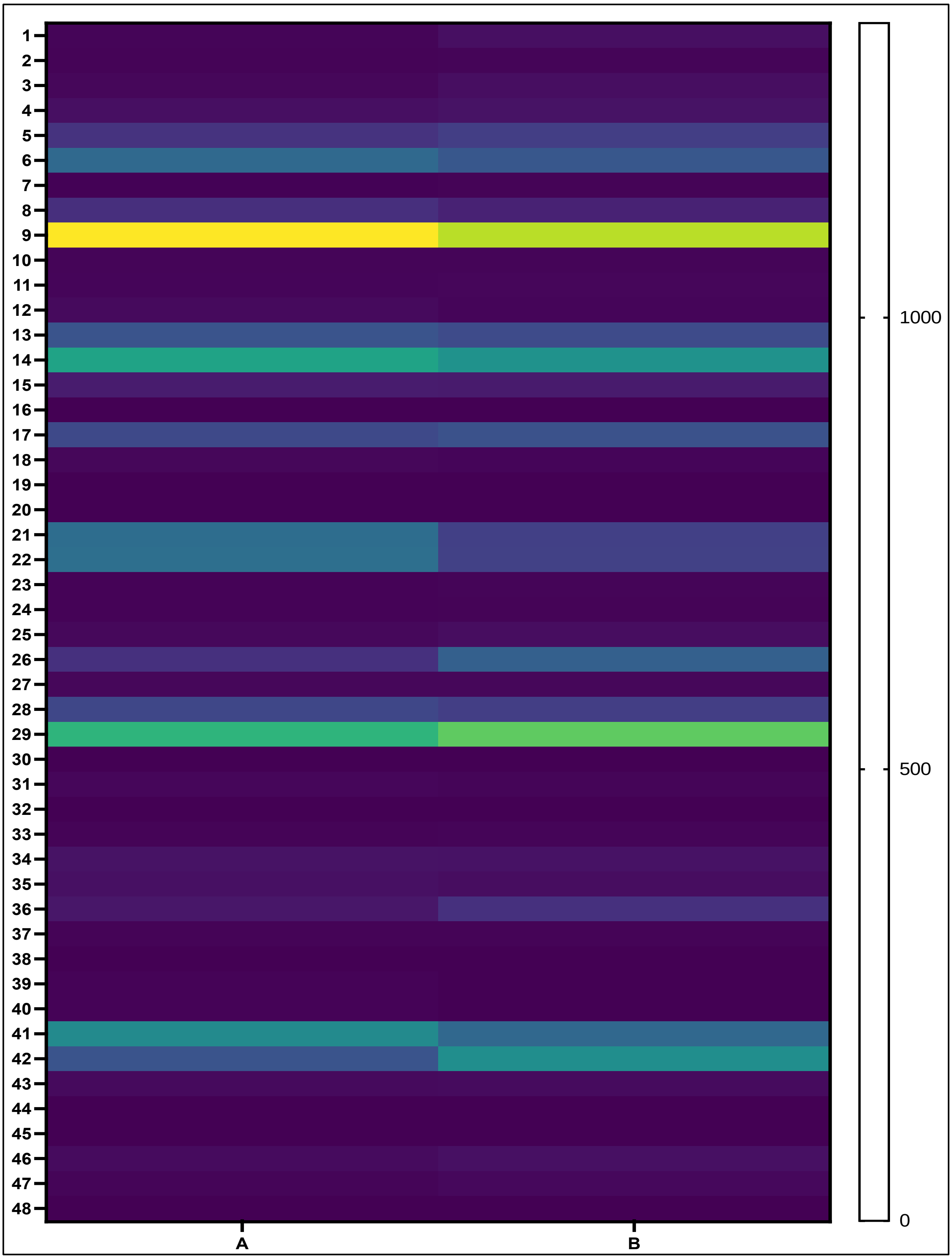

Patient 6. P < 0.0001

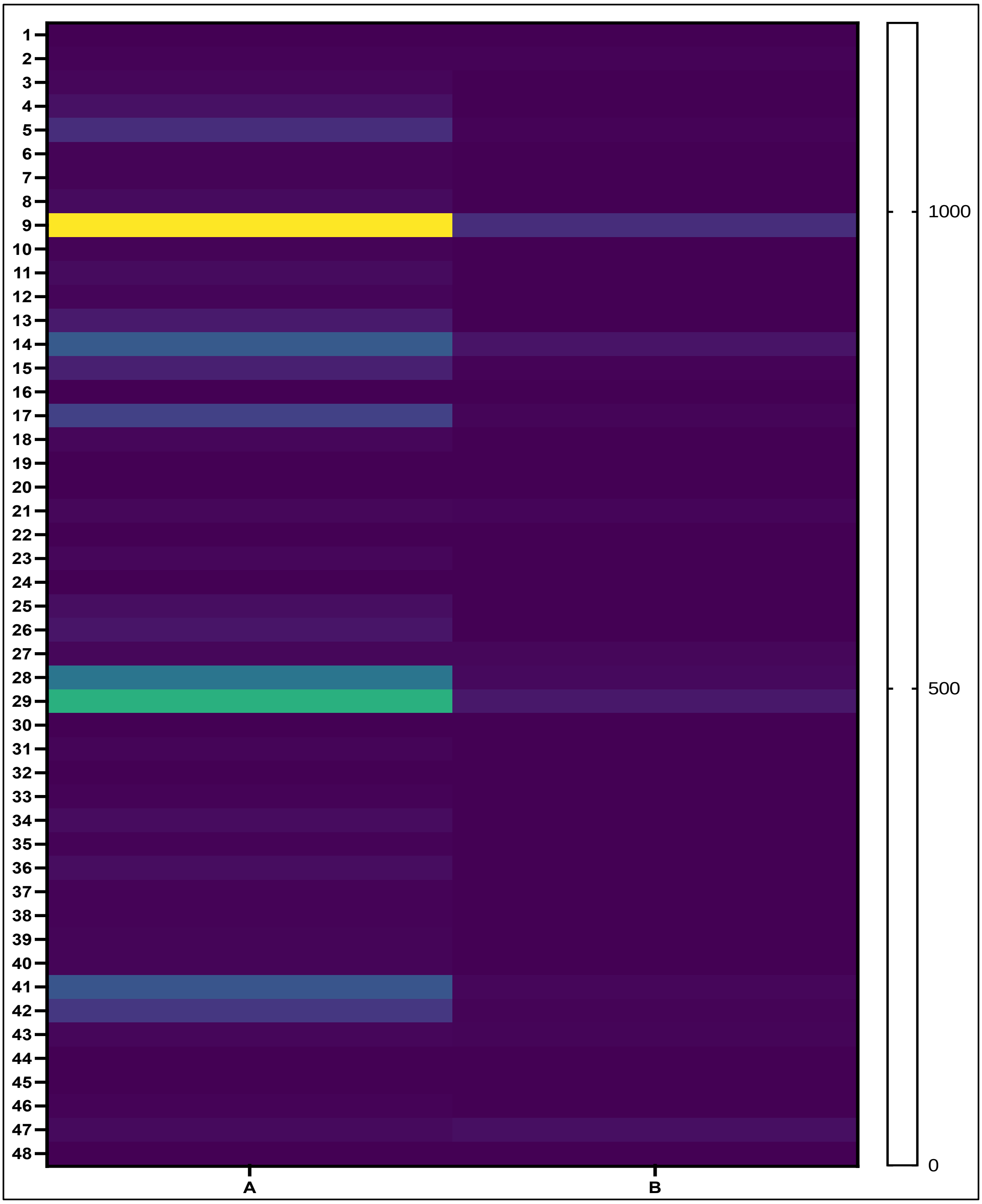

Patient 7. P = 0.0007

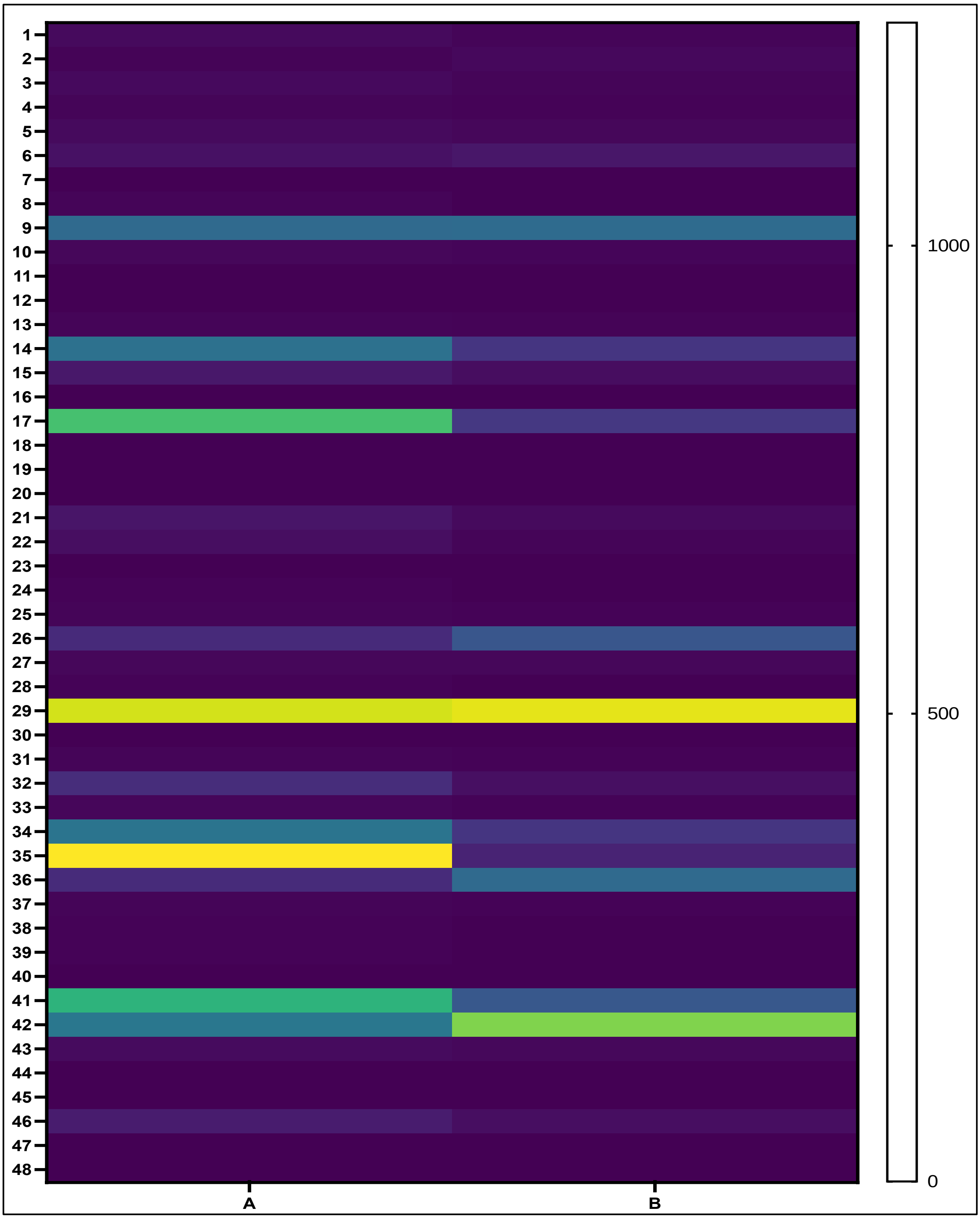

Patient 8. P =0.44

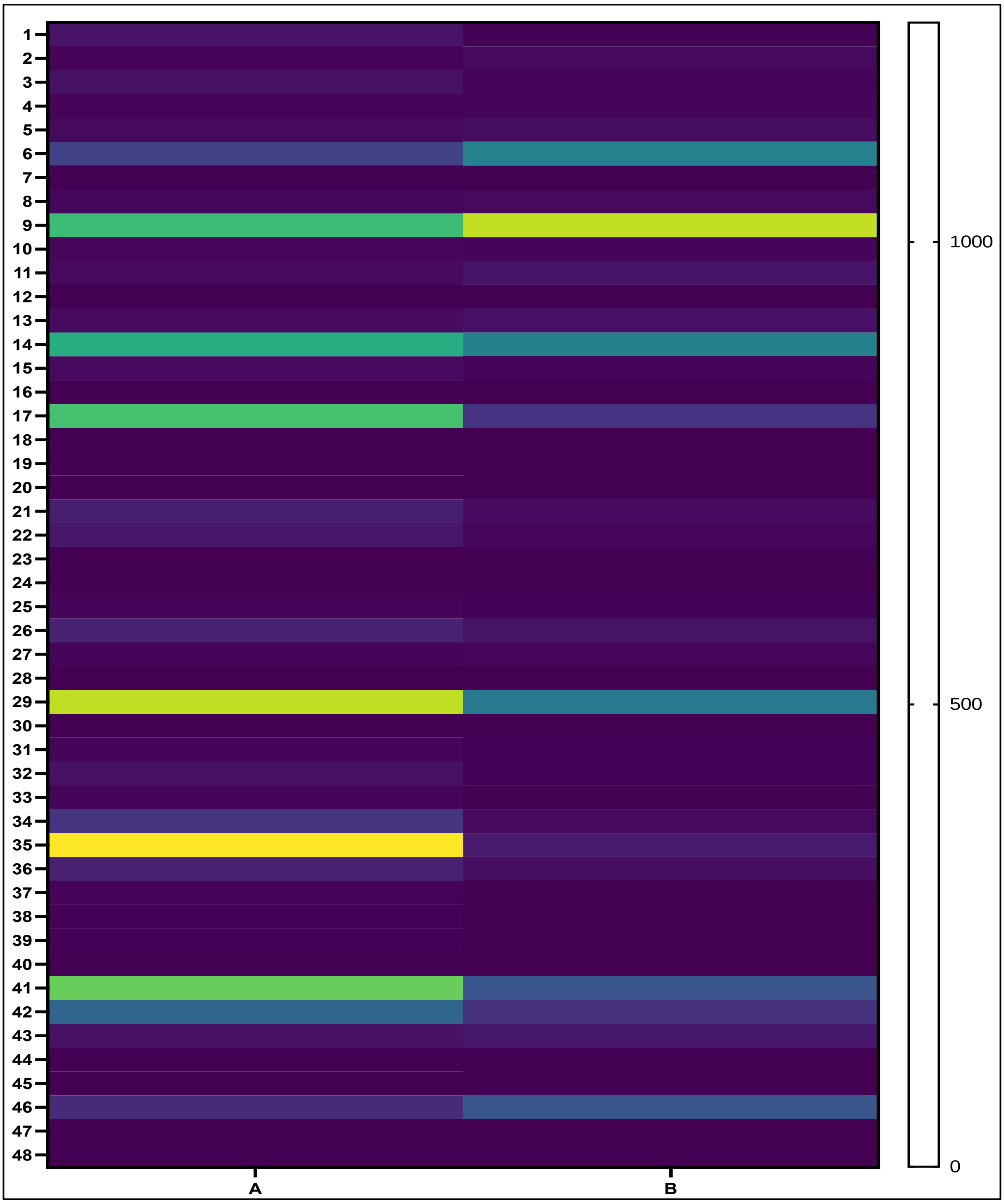

Patient 9. P < 0.0001

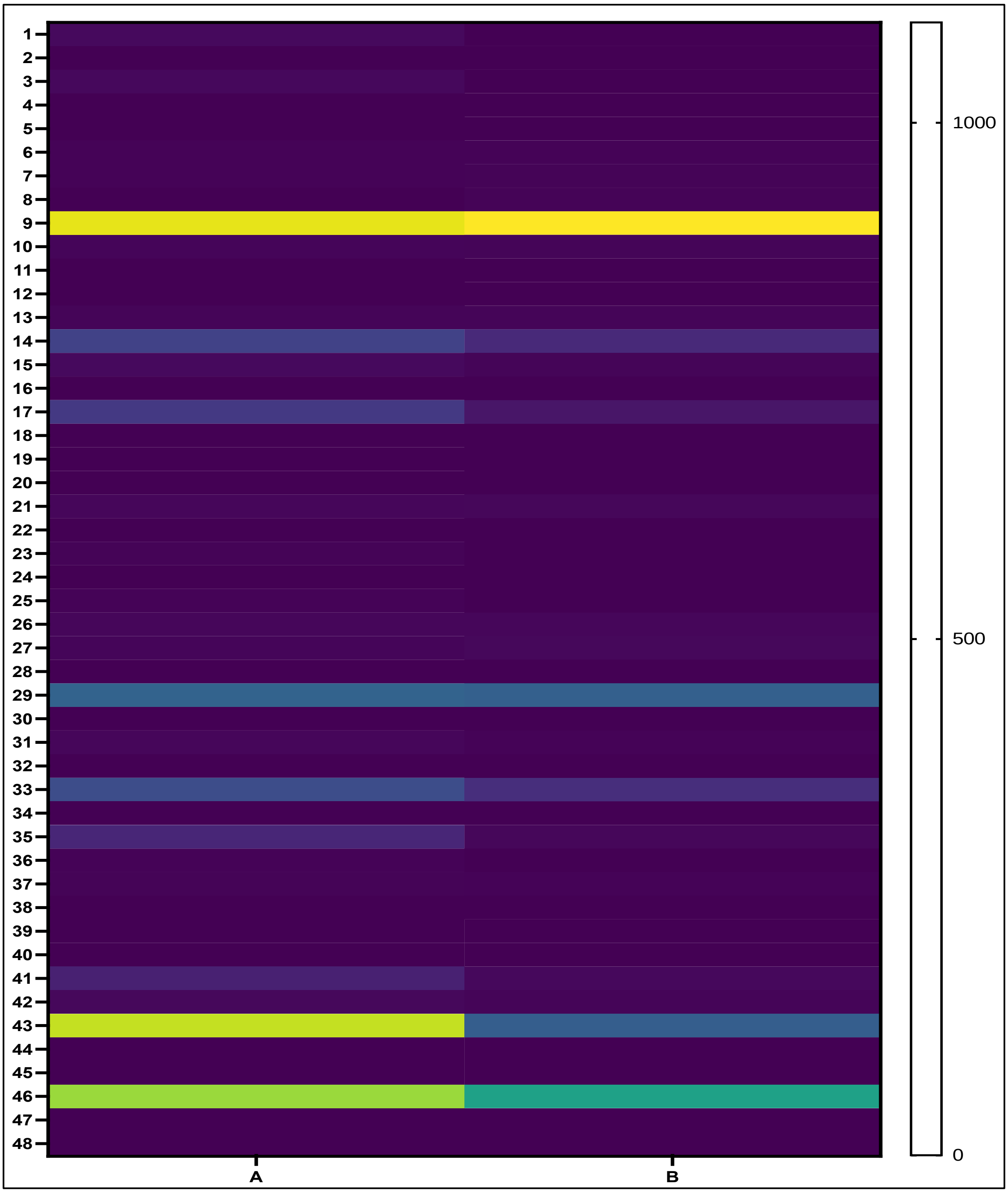

Patient 10. P < 0.0001

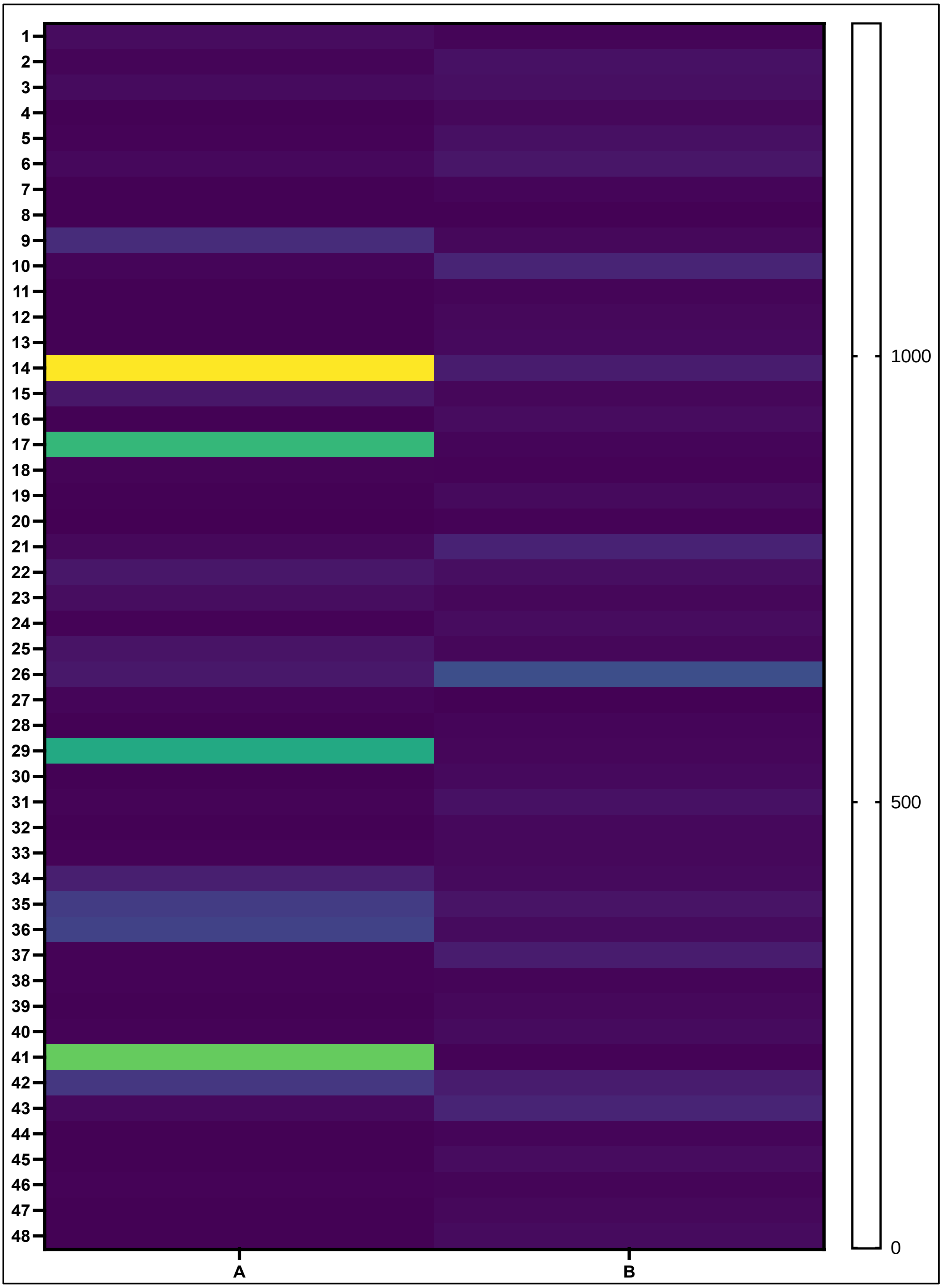

Patient 11. P = 0.19

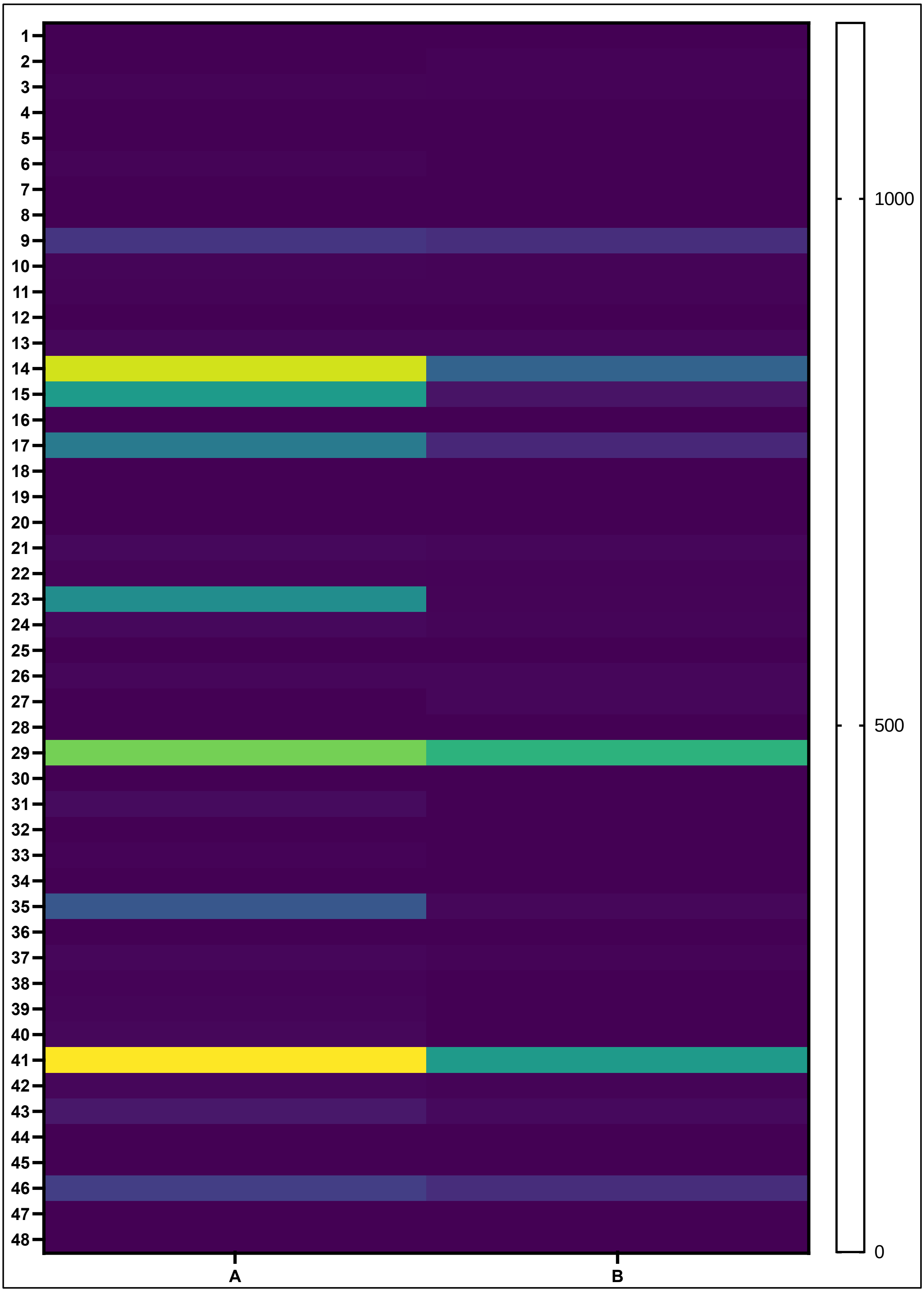

Patient 12. P = 0.12

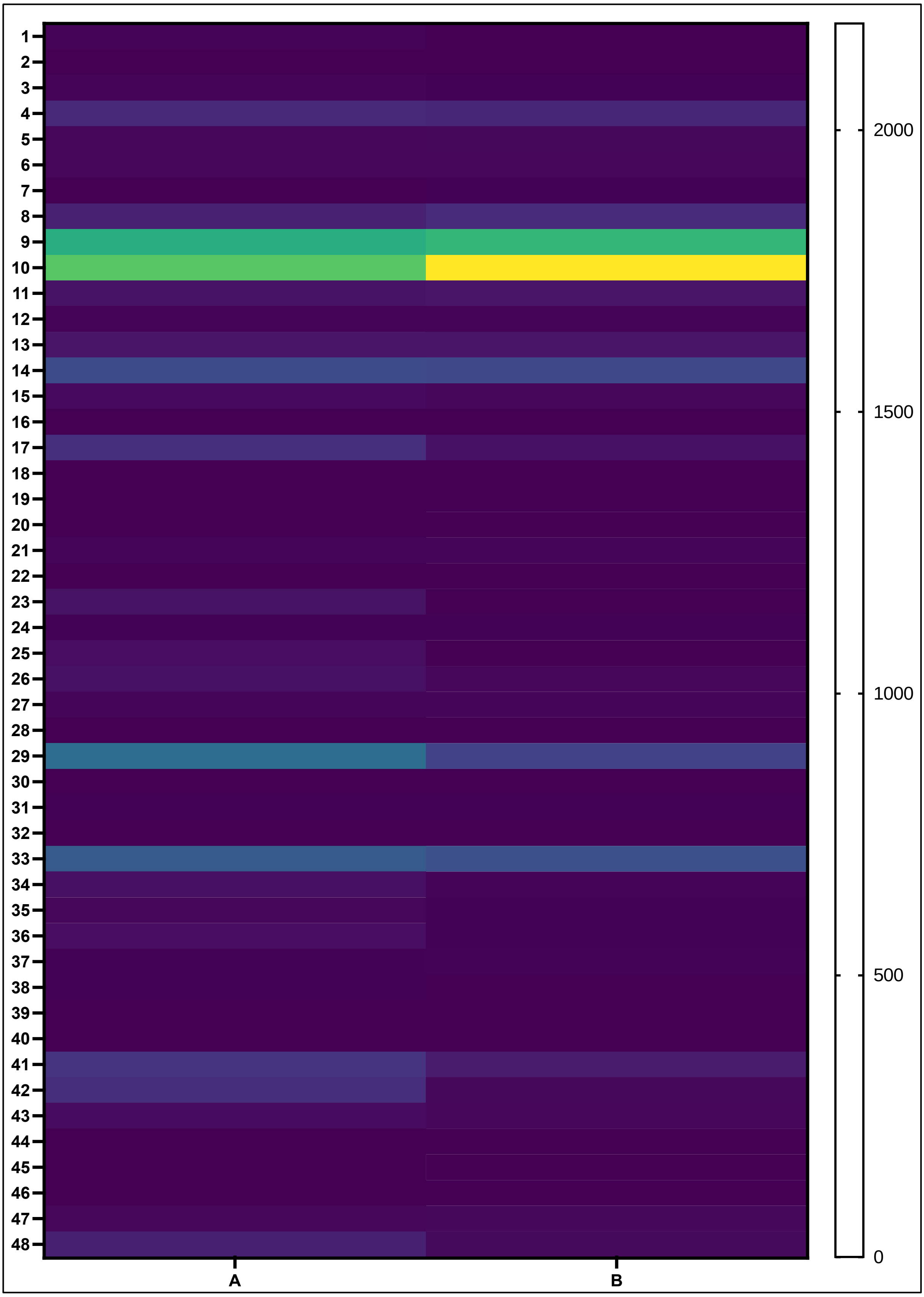

Patient 13. P = 0.014

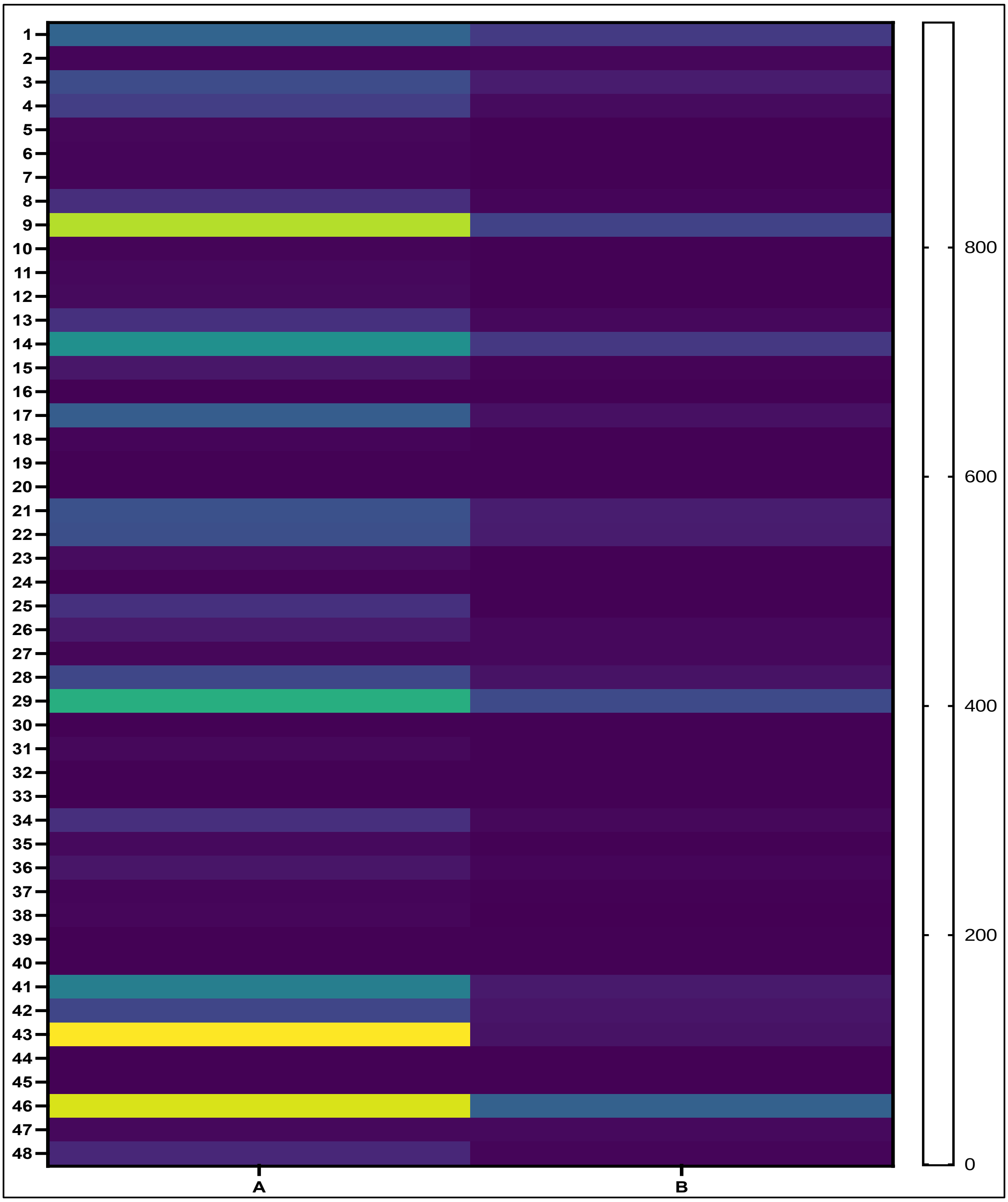

Patient 14. P = 0.03

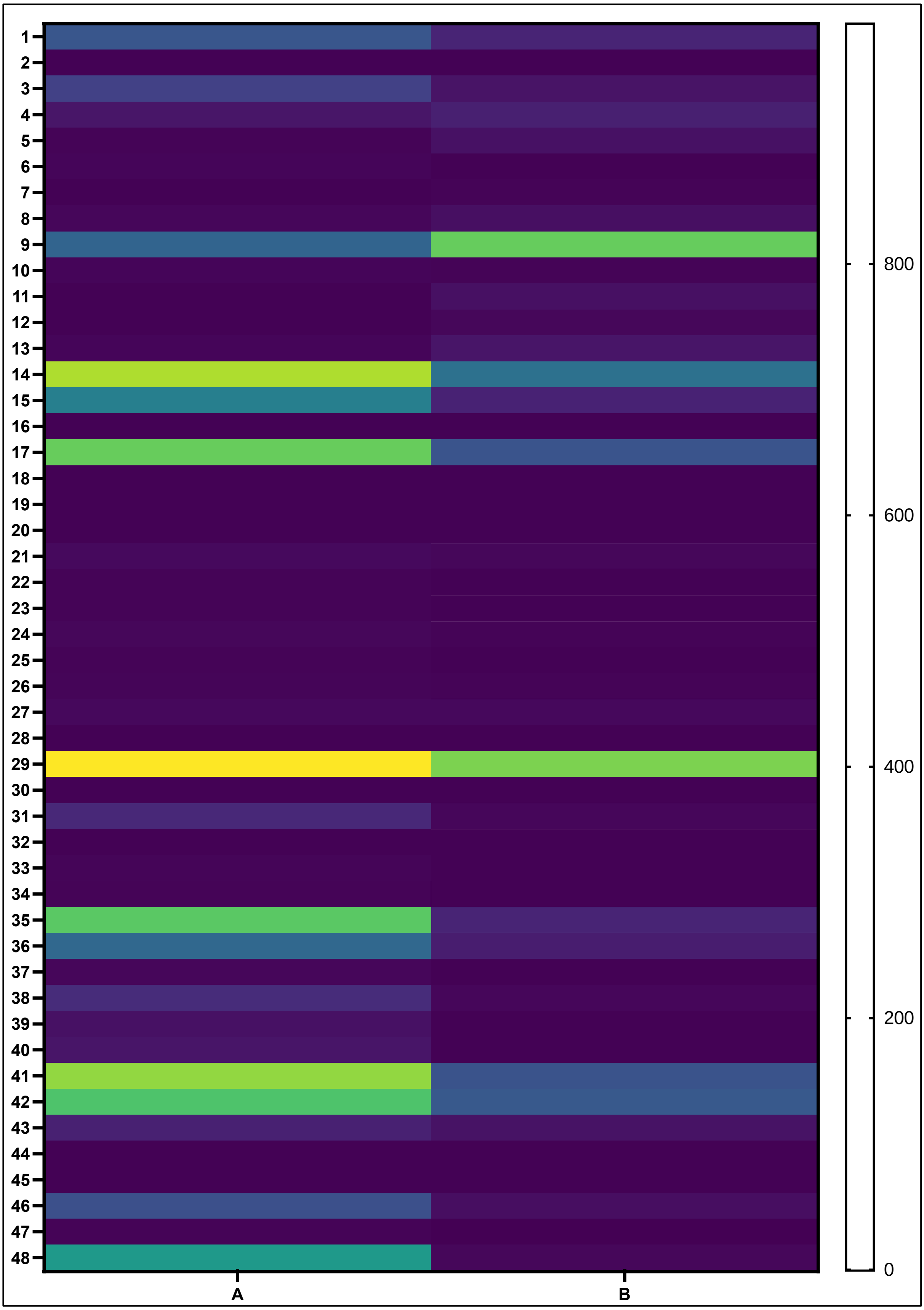

Patient 15. P = 0.0016

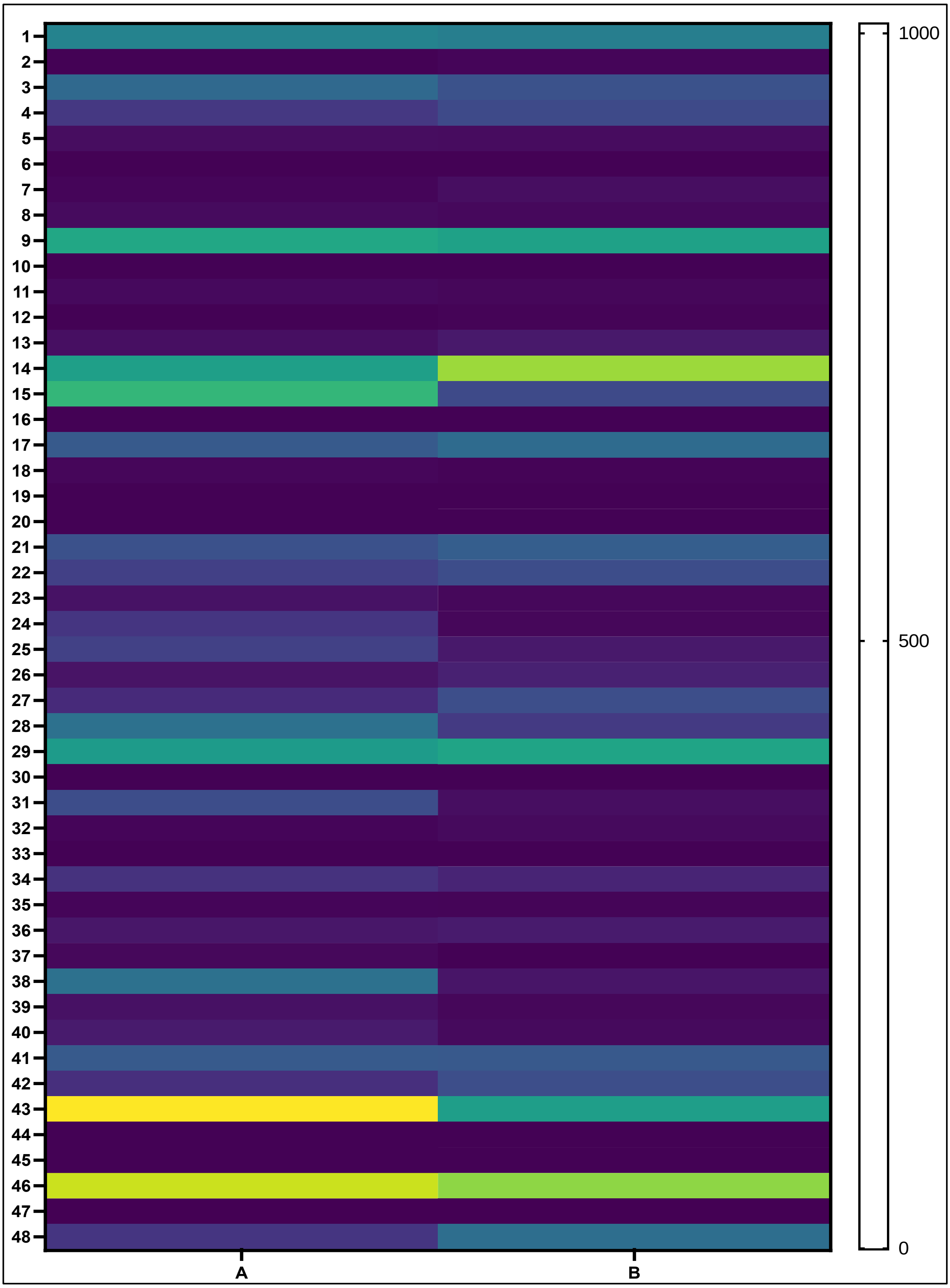

Patient 16. P = 0.54

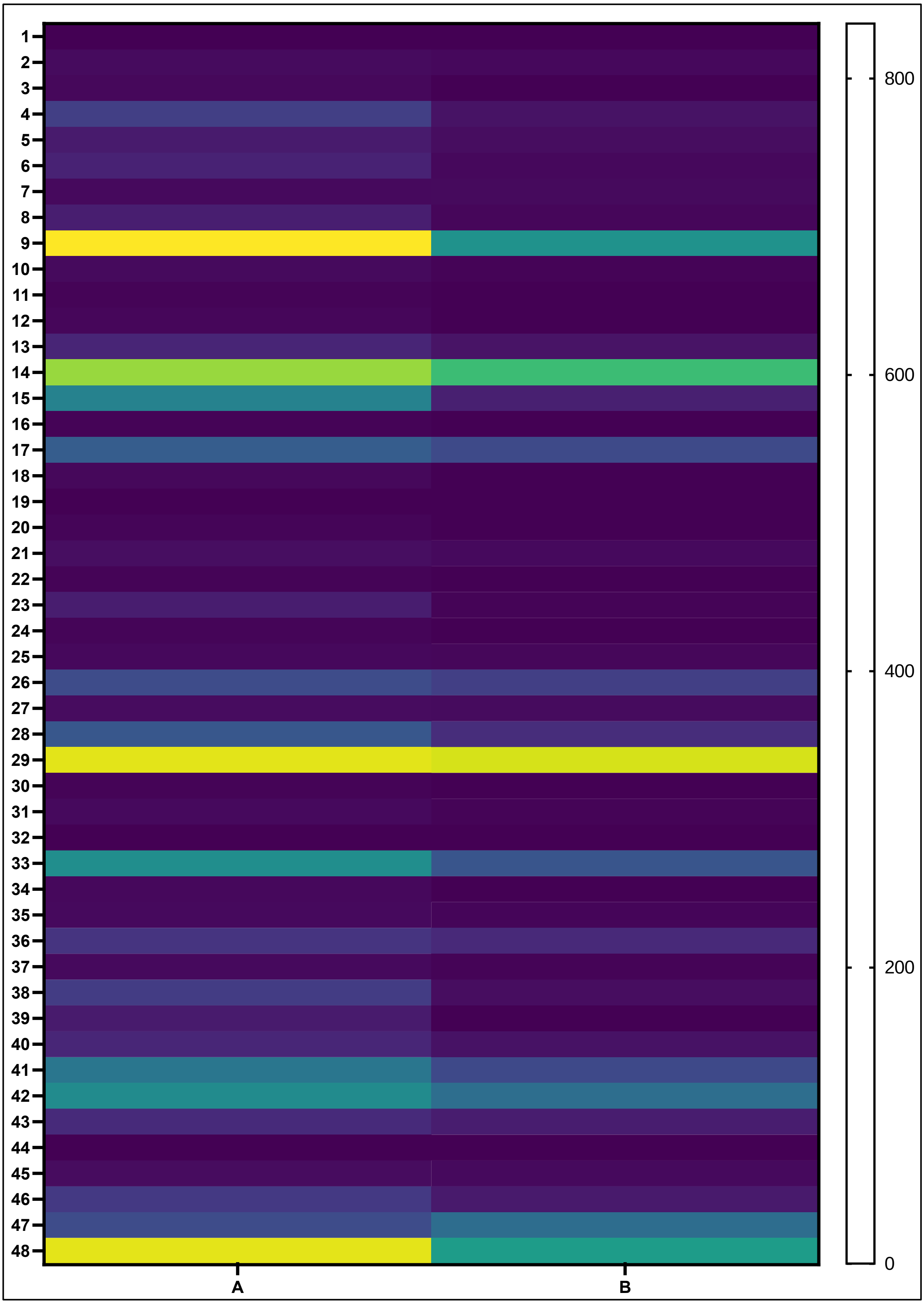

Patient 17. P < 0.0001

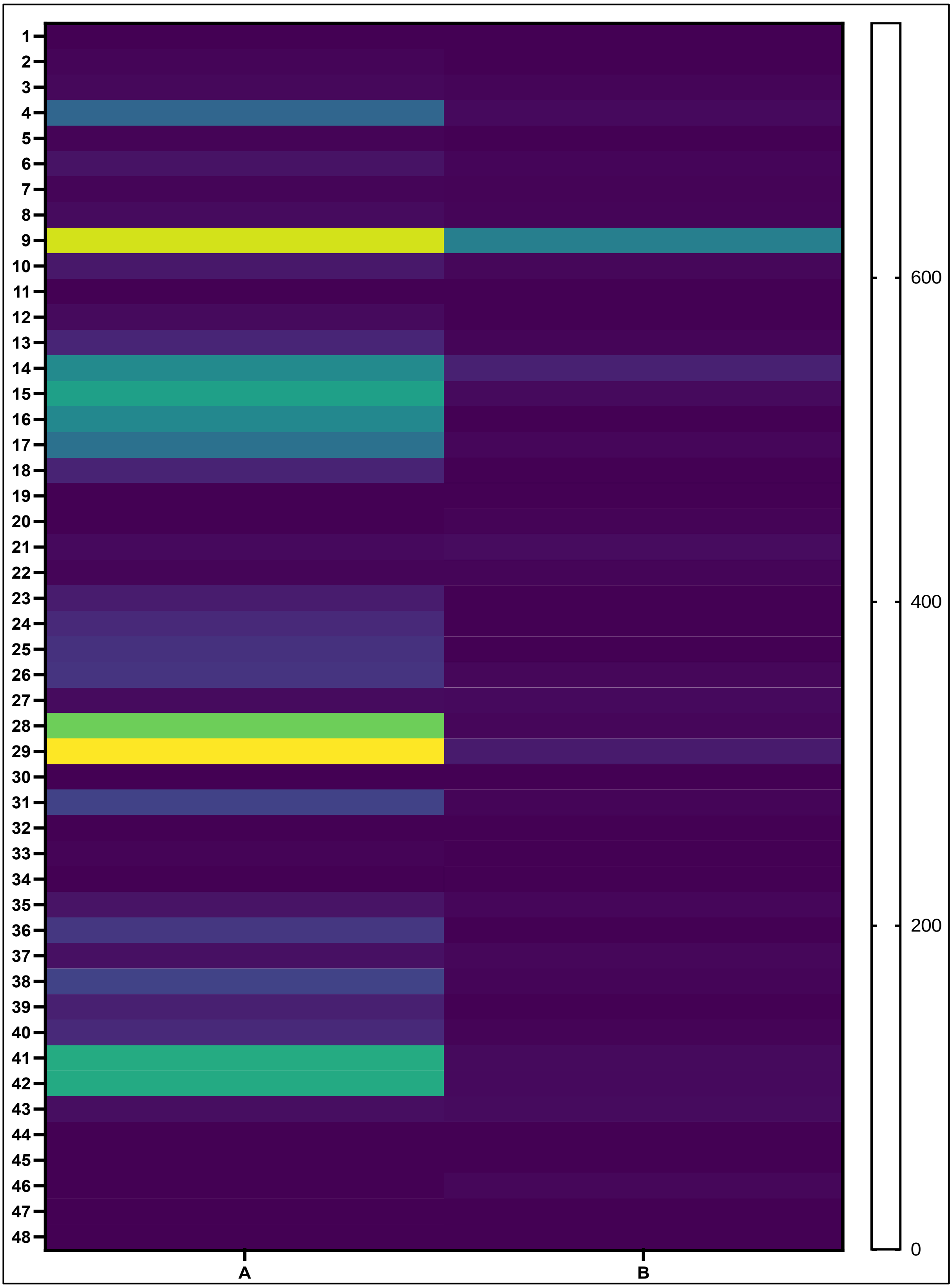

Patient 18. P = 0.015

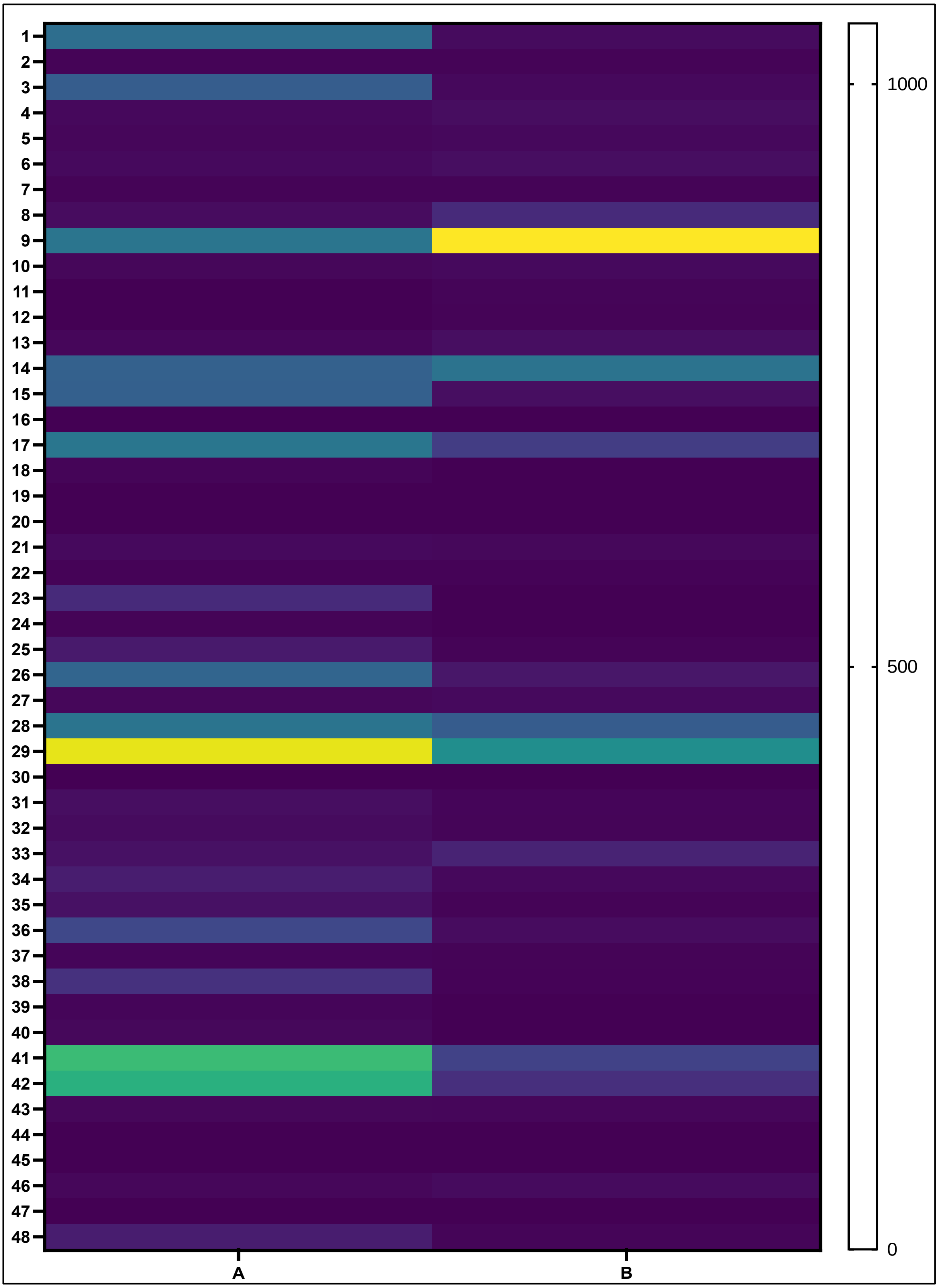

Patient 19. P = 0.01

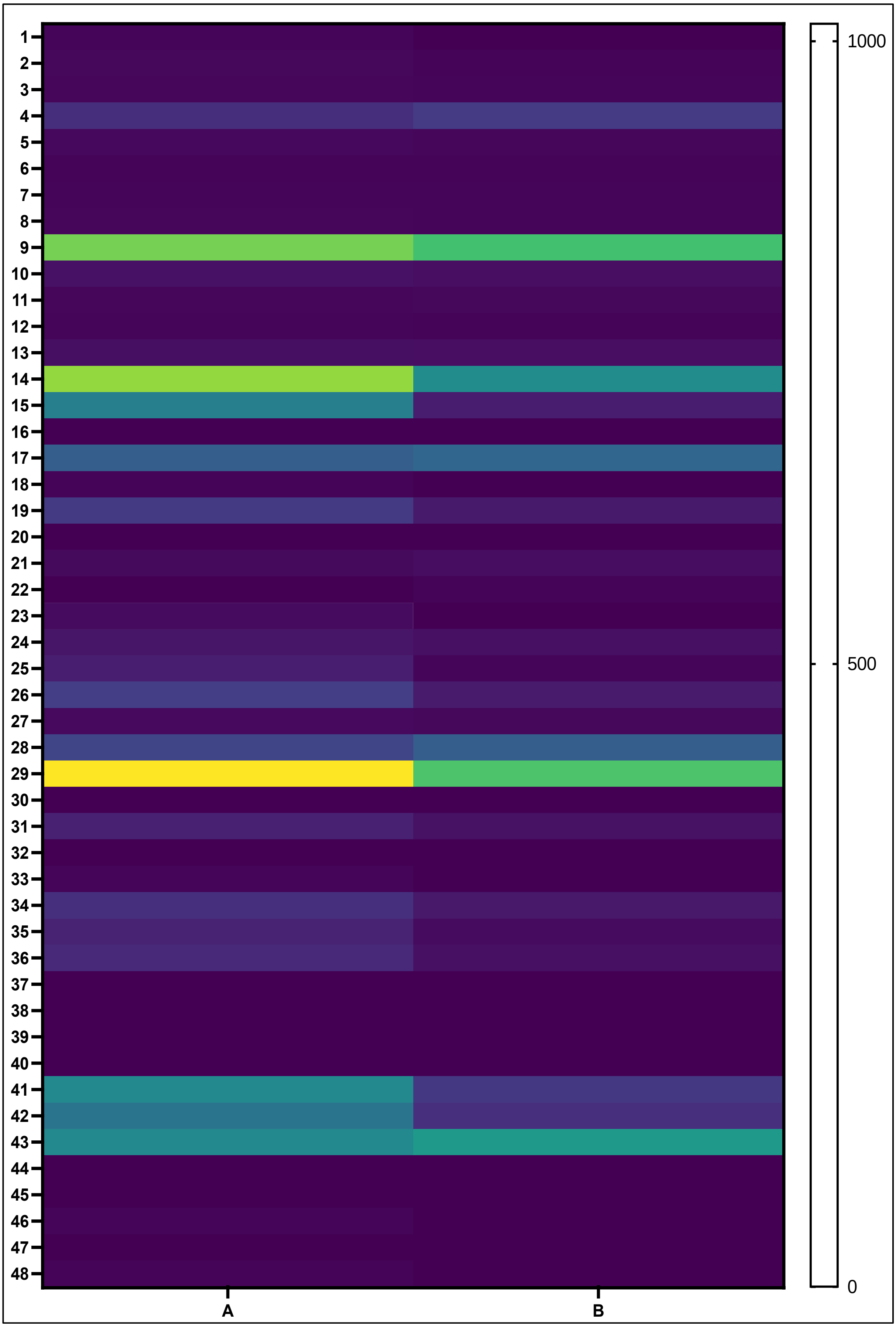

Patient 20. P = 0.0006

Supplementary References:

1. Vebo, H.C., et al., *Temporal development of the infant gut microbiota in immunoglobulin E-sensitized and nonsensitized children determined by the GA-map infant array.* Clin Vaccine Immunol, 2011. **18**(8): p. 1326-35.
